# Landscape of RNAs in human lumbar disc degeneration

**DOI:** 10.18632/oncotarget.11334

**Published:** 2016-08-17

**Authors:** Ping-Heng Lan, Zhi-Heng Liu, Yan-Jun Pei, Zhi-Gang Wu, Yang Yu, Yong-Feng Yang, Xu Liu, Lu Che, Chi-Jiao Ma, Yan-Ke Xie, Qing-Jie Hu, Zhong-Yuan Wan, Hai-Qiang Wang

**Affiliations:** ^1^ Department of Orthopaedics, Xijing Hospital, Fourth Military Medical University, Xi'an, P. R. China; ^2^ Department of Orthopaedics, Xi'an Air Force Hospital, Xi'an, P.R. China; ^3^ The Second Department of Surgery, No. 518 Hospital of the PLA, Xi'an, P. R. China; ^4^ Department of Orthopaedic Surgery, Lanzhou General Hospital of Lanzhou Military Region, PLA, Lanzhou, P. R. China; ^5^ State Key Laboratory of Military Stomatology, Department of Periodontology, School of Stomatology, Fourth Military Medical University, Xi'an, P. R. China; ^6^ Shaanxi Key Laboratory of Stomatology, Biomaterials Unit, School of Stomatology, Fourth Military Medical University, Xi'an, P. R. China; ^7^ Company Eleven, Brigade of Undergraduates, Fourth Military Medical University, Xi'an, P. R. China; ^8^ Aerospace Medical School, Fourth Military Medical University, Xi'an, P. R. China; ^9^ The First Hospital of Hanbin Area, Ankang, P. R. China; ^10^ Department of Orthopedics, General Hospital of Beijing Military Command, Beijing, P. R. China; ^11^ Department of Orthopaedics, No. 5 Hospital of the PLA, Yinchuan, P. R. China

**Keywords:** noncoding RNAs, miRNAs, circRNAs, gene regulation, RNA transcription

## Abstract

Accumulating evidence indicates noncoding RNAs (ncRNAs) fine-tune gene expression with mysterious machinery. We conducted a combination of mRNA, miRNA, circRNA, LncRNA microarray analyses on 10 adults' lumbar discs. Moreover, we performed additional global exploration on RNA interacting machinery in terms of *in silico* computational pipeline. Here we show the landscape of RNAs in human lumbar discs. In general, the RNA-abundant landscape comprises 14,635 mRNAs (37.93%), 2,059 miRNAs (5.34%), 18,995 LncRNAs (49.23%) and 2,894 (7.5%) circRNAs. Chromosome 1 contributes for RNA transcription at most (10%). Bi-directional transcription contributes evenly for RNA biogenesis, in terms of 5′ to 3′ and 3′ to 5′. Despite the majority of circRNAs are exonic, antisense (1.49%), intergenic (0.035%), intragenic (1.69%), and intronic (6.29%) circRNAs should not be ignored. A single miRNA could interact with a multitude of circRNAs. Notably, CDR1as or ciRS-7 harbors 66 consecutive binding sites for miR-7-5p (previous miR-7), evidencing our pipeline. The majority of binding sites are perfect-matched (78.95%). Collectively, global landscape of RNAs sheds novel insights on RNA interacting mechanisms in human intervertebral disc degeneration.

## INTRODUCTION

Intervertebral disc degeneration (IDD) is the chief contributing factor to low back pain with deleterious medical and social impact [1]. Increasing evidence reveals that IDD is a multifaceted spinal disease. Both genetic and environmental factors contribute to IDD, the chief of which are genetics [2, 3]. The genetics machinery ranges from single-nucleotide variants [4] and coding genes [5–8], to newly defined noncoding RNAs (ncRNAs).

Recent whole-genome sequencing (WGS) studies have greatly expanded our scopes upon human genome [9, 10]. As a multitude set of transcript products of the human genome, ncRNAs account for 98% of the human genome devoid of protein-coding function. Overwhelming evidence indicates that ncRNAs widely exist in living things. Moreover, ncRNAs play critical roles in a variety of biological processes pertaining to gene expression [11, 12].

The ncRNA superfamily comprises small ncRNAs (miRNAs), long ncRNAs (LncRNAs) and the newly defined circular RNAs (circRNAs) in terms of length and structure. Indeed, RNAs play fundamental roles in organisms. Not only different lengths, but sequences or structures (e.g., riboswitches) of RNAs, are critical [13, 14]. In structure, miRNAs are ~21-nucleotide-long noncoding RNAs. LncRNAs contain nucleotides over 200, even more than 100,000 [15]. CircRNAs are a class of ncRNAs with covalent linked ends. Emerging as the first representative of ncRNA family, miRNAs direct an effector protein Argonaute (AGO) to suppress their targets' expression [16]. The mysterious class of LncRNAs has been addressed recently with increasing uncovered numbers. Furthermore, a growing body of evidence indicates that LncRNAs play important roles in each issue of human biology [17]. LncRNAs can be divided into different subgroups in terms of their genomic contexts: stand-alone LncRNAs or lincRNAs [18]; Natural antisense transcripts [19]; Long intronic ncRNAs [20]; Divergent transcripts, promoter-associated transcripts and enhancer RNAs [21]. In mechanism, LncRNAs are key regulators in epigenetics as recruiters, tethers and scaffolds [22]; in transcription as decoys, coregulators and Pol II inhibitors [23]; in post-transcriptional regulation involving in mRNA processing, stability and translation [23].

In contrast, studies on circRNAs are at their early stage. It has been noted that circRNAs act as post-transcriptional regulators. CircRNA expression is tissue/developmental stage specific. They can interact with miRNAs via miRNA sponges and competing endogenous RNA (ceRNA) in the cytoplasm [24–26]. In details, miRNA sponges indicate that a circRNA with miRNA binding sites could absorb the miRNA and eliminate the original repression on the miRNA-targeted gene.

Regarding ncRNAs in human IDD, we have addressed the expression profiles of miRNAs previously using scoliotic nucleus pulposus (NP) as control [27]. Moreover, we addressed the expression profiling of LncRNAs and mRNAs in IDD using normal NP control [28]. It has been noted that there might be a regulatory link between LncRNAs, circRNAs and miRNAs. LncRNAs and circRNAs could repress miRNAs via various mechanisms, e.g. miRNA sequestration. Furthermore, the interaction of circRNAs and LncRNAs remains mysterious.

On the other hand, it should be stressed that discs from scoliotic patient are abnormal [29]. Accordingly, we conducted miRNAs and circRNAs microarray analyses using the same total RNA samples from lumbar NP tissues as LncRNA-mRNA microarray. Moreover, advanced bioinformatics data analyses were employed.

## RESULTS

### SuperSeries of human RNAs in lumbar disc samples

**Figure 1:**
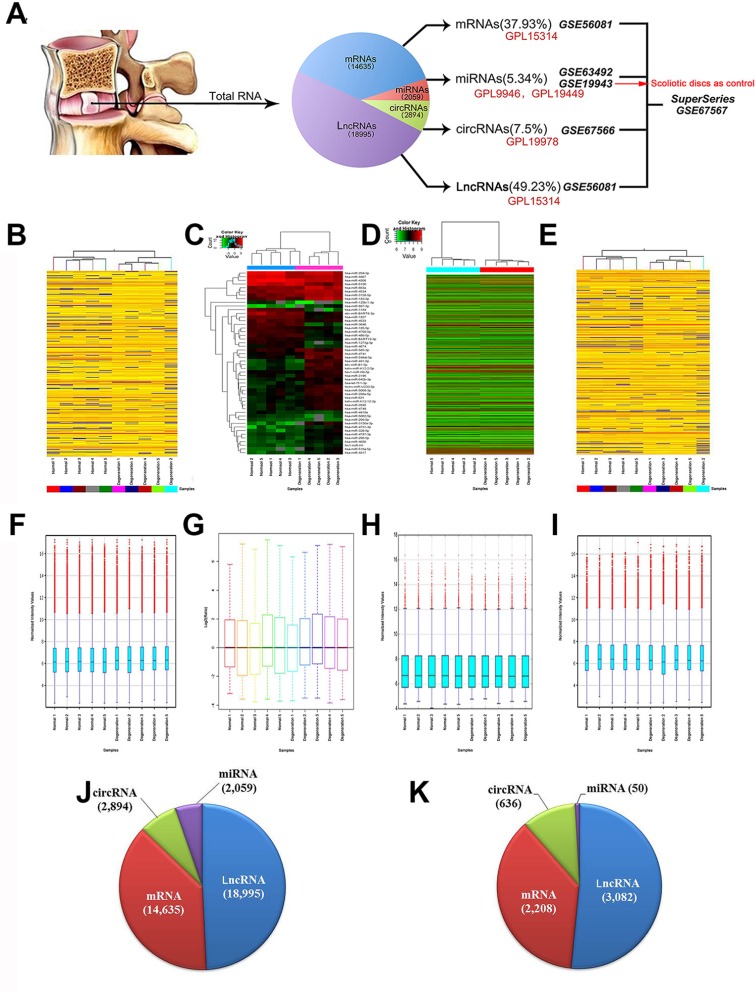
SuperSeries of ncRNAs in human lumbar discs (**A**) Schematic diagram of human lumbar disc RNA SuperSeries based on tetrad platforms. (**B**–**E**) delineate the Hierarchical clustering of mRNAs, miRNAs, circRNAs and lncRNAs; whereas (**F**–**I**) represent the boxplots of mRNAs, miRNAs, circRNAs and lncRNAs. (**J**) indicates the ratio of RNA subgroups in human lumbar discs. (**K**) indicates the ratio of differentially expressed RNA subgroups in human IDD.

Based on tetrad array platforms using the same 10 lumbar disc samples, we successfully established a SuperSeries in GEO (GSE67567) [28, 30] (Figure 1A). The BioProject accession of this SuperSeries is PRJNA280271, which encompasses and links the expression profiles of miRNAs (GSE63492), LncRNAs and mRNAs (GSE56081) and circRNAs (GSE67566). We addressed RNAs expression profiles by comparing RNAs in 5 degenerate disc samples with those in healthy disc samples.

### Landscape of RNAs in human IDD

In general, human lumbar discs are RNA-abundant, the expression profiling of which comprise 14,635 mRNAs out of 30215 protein-coding transcripts (37.93%, Supplementary Table S1), 2,059 miRNAs (5.34%, Supplementary Table S2), 18,995 LncRNAs (49.23%, Supplementary Table S3) and 2,894 circRNAs (7.5%, Supplementary Table S4). mRNAs account for 37.93% of total RNAs; whereas ncRNAs account for 62.07% of total RNAs (Figure 1J). mRNAs and LncRNAs account for the majority of RNA splicing products (87.16%).

Figure 1B–1E delineated the Hierarchical clusterings of mRNAs, miRNAs, circRNAs and LncRNAs; whereas Figure 1F–1I represented the boxplots of mRNAs, miRNAs, circRNAs and LncRNAs.

As for differentially expressed RNAs, there were 2,208 mRNAs (36.35%, Supplementary Table S5), 50 miRNAs (2.45%, Supplementary Table S6), 3,082 LncRNAs (50.73%, Supplementary Table S7) and 636 circRNAs (10.47%, Supplementary Table S8) reached statistical threshold (Figure 1K).

### Global analysis reveals RNA transcription and binding truth

Functional annotation generated 3,146 potentially interactive networks of differentially expressed circRNAs and miRNAs in terms of computational pipeline (Supplementary PDF S1).

**Figure 2:**
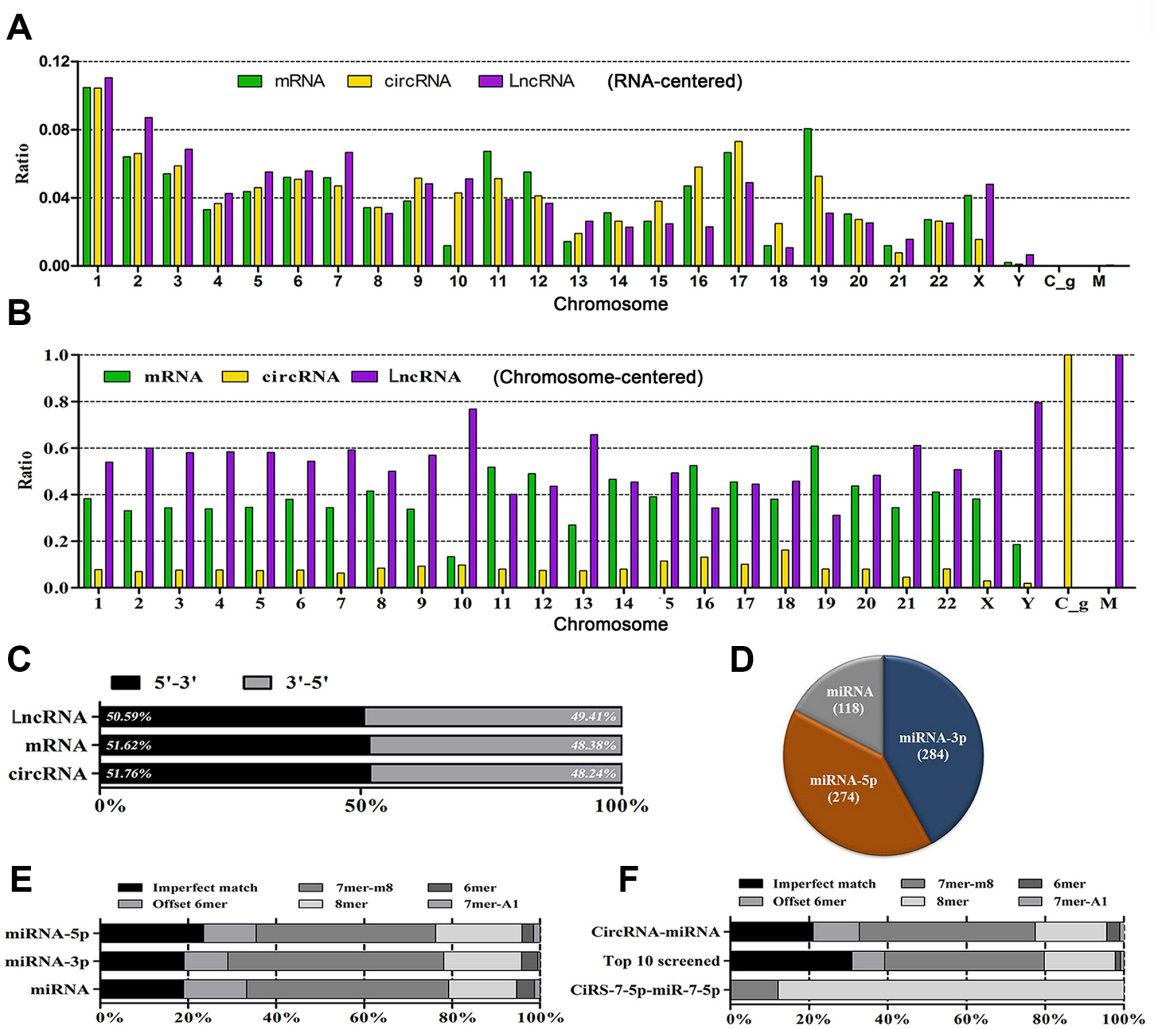
The landscape of RNAs and their binding sites (**A**) Represents the distribution diagram of mRNAs, circRNAs and LncRNAs in each Chromosome in terms of each type of RNA; whereas (**B**) represents the constituent ratio of mRNAs, circRNAs and LncRNAs in each Chromosome in terms of Chromosome (normalized value = 1.0). (**C**) delineates the constituent ratio of transcription direction of RNAs. (**D**) indicates the constituent ratio of miRNAs interacting with circRNAs. (**E**) represents the hallmarks of binding sites in terms of miRNA types; whereas (**F**) represents the features of binding sites in terms of global, top 10 differentially expressed and exceptional views.

Chromosome 1 transcribes RNAs at the highest level (10%); whereas Chromosome Y contributes the least. For circRNA transcription, Chromosome 17 ranks as the second candidate, following by Chromosome 2. For mRNA transcription, Chromosome 19 ranks as the second one, following by Chromosome 11. For LncRNAs, the ranking order is Chromosome 2 and 3 (Figure 2A, 2B, normalized value = 1). Bi-direction transcription contributes nearly equally for RNA biogenesis in terms of 5′ to 3′ and 3′ to 5′ (Figure 2C).

### Binding sites hallmarks

In total, there were 5,310 binding sites between circRNAs and miRNAs. miRNAs interact with circRNAs via miR-5p (45.07%), miR-3p (36.37%), and miR *per se* (18.57%) (Figure 2D). The majority of binding sites are perfect-matched (78.95%), with imperfect match as 21.05% (Figure 2E, 2F). The binding site feature is similar in terms of top 10 differentially expressed miRNAs and circRNAs. Moreover, the classic CiRS-7-5p binds with miR-7-5p via 100% perfect-match (Figure 2F).

### Interacting hallmarks from the point of miRNAs

**Figure 3:**
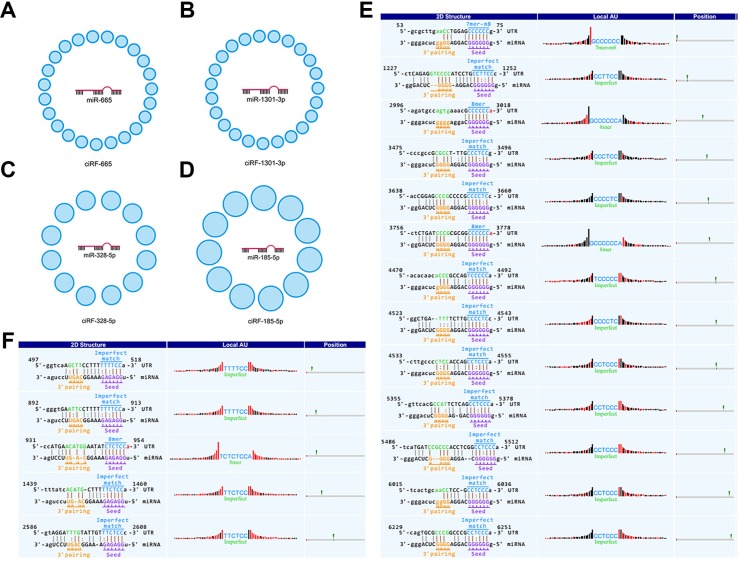
circRNA families interacting with miRNAs (**A**–**D**) delineate circRNA families binding with corresponding miRNAs. (**E** and **F**) indicate exceptional cases of multiple binding sites between circRNAs and miRNAs.

We found that a single miRNA could interact with a multitude of circRNAs. Typically, miR-665 interacts with 23 circRNAs. Accordingly, we termed these circRNAs as ciRF-665 (circRNA family binding with miR-665, Figure 3A). Similarly, we found ciRF-1301-3p (circRNA family binding with miR-1301-3p) with 23 members (Figure 3B), ciRF-328-5p (circRNA family binding with miR-328-5p) with 12 members (Figure 3C), cirRF-185-5p (circRNA family binding with miR-185-5p) with 11 members (Figure 3D).

All reported miRNA sponges, circRNAs, have only one or two binding sites for the same miRNA. The extreme case is miR-7 and its sponge, CiRS-7, with over 70 conserved binding sites [31], or CDR1 as [32] as indicated by circBase [33] and CircInteractome [34]. Notably, we found that CiRS-7 or CDR1as (hsa_circ_0001946) harbors 66 consecutive binding sites for miR-7-5p (previous miR-7). Therefore, the line of evidence greatly validated our computational pipeline. Moreover, FCHO1as (circRNA-001396), antisense to *FCHO1*, harbors 13 binding sites for miR-328-5p (Figure 3E); whereas IL4Rex (circRNA-000684), exonic spliced from *IL4R*, harbors 5 binding sites for miR-185-5p (Figure 3F).

### Interacting hallmarks from the point of circRNAs

**Figure 4:**
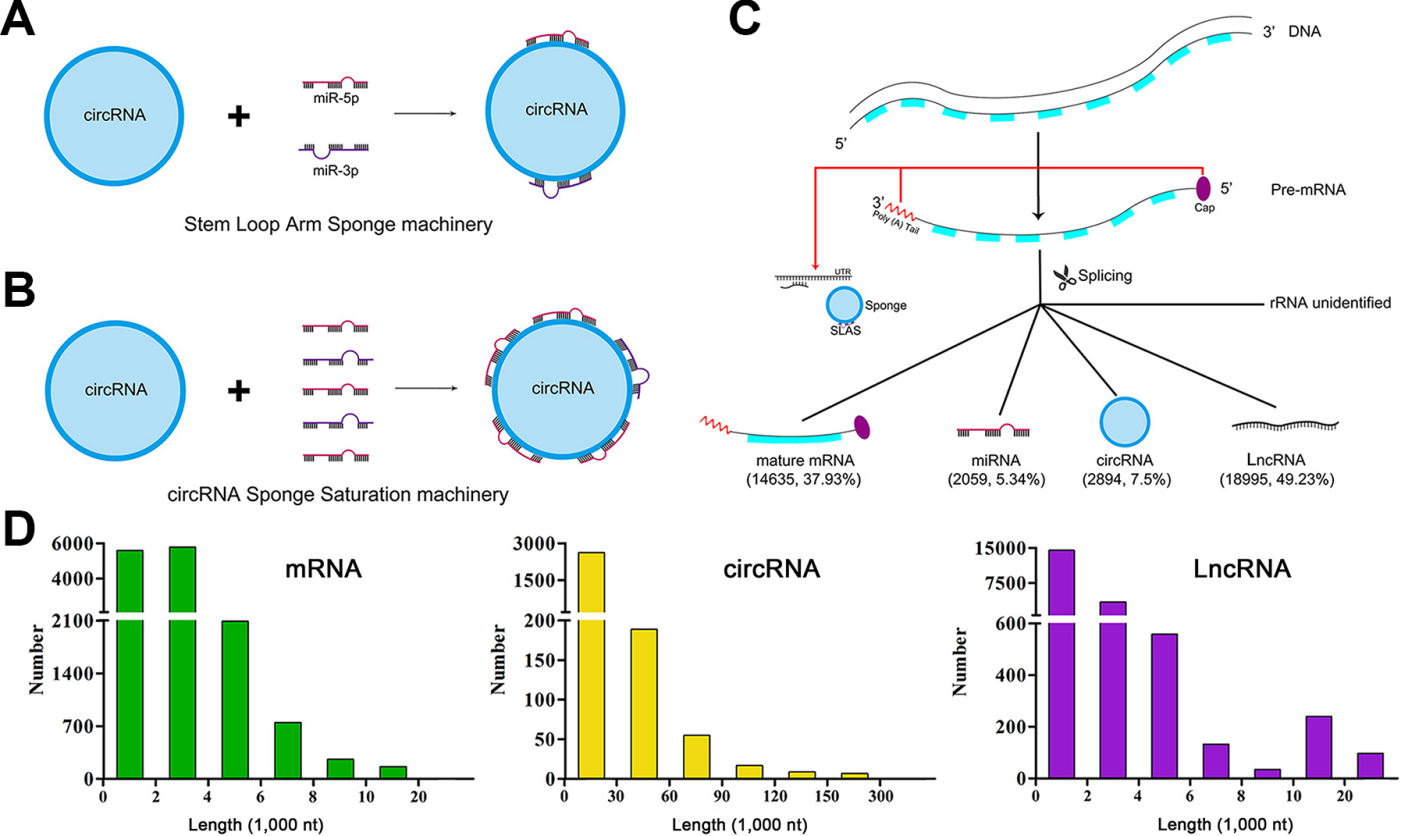
The miRNA-circRNA binding machinery and landscape of RNA length (**A**–**B**) indicate miRNA-circRNA interacting machinery. (**C**) is the diagram of RNA transcription and splicing. (**D**) delineates the length scope of each type of RNAs.

We noted that circRNAs commonly combined with the 3p or 5p of miRNA (Supplementary Table S9). It is well established that miR-3p or miR-5p derives from the arms of stem loops of pre-miRNAs. We termed the phenomena as Stem Loop Arm Sponge (SLAS, Figure 4A). Moreover, we found that a single circRNA commonly interact with at most 5 miRNAs (99.31%) as sponges following intensive exploration. In total, there were 20 circRNAs interacting with less than 5 miRNAs (range 1–4, Supplementary Table S10). Considering the available evidence [26] and the principle of complementary base pairing, we putatively put forward the phenomena as sponge saturation (Figure 4B, Supplementary Figure S1).

### Binding principle for ncRNAs and mRNAs

It is well established that RNAs have polarity with one 3′ ends and one 5′ ends. At the 3′ ends, most eukaryotic mRNAs have a sequence of polyadenylic acid, referred to as the poly (A) tail. Appropriate poly (A) tail length is crucial for efficient translation, being ~70 residues [35, 36]. As well, the methyl guanosine ‘cap’ at the 5′ ends of all eukaryotic mRNAs plays critical roles during mRNA processing and metabolism [37].

Pre-RNAs undergo several steps of RNA splicing during which the phosphodiester bonds at exon-intron boundaries are cleaved and the introns are excised. The process is transcription during which RNAs mature. circRNAs are chiefly the products of exons splicing co-transcriptionally mediated by the spliceosome and flanking introns [24].

Furthermore, one RNA binds with another RNA in accordance with the polarity principle. In details, when a single miRNA meets its targeted mRNA, the 3′ end of the miRNA binds with the 5′ end of the mRNA; whereas the 5′ end of the miRNA binds to the 3′ end of the mRNA.

Traditionally, miRNAs are reported to repress gene expression by binding with the seed regions of the 3′UTRs of their targeted genes. Accumulating evidence suggests that miRNAs can suppress gene expression by complementary interactions with the coding sequence (CDS) and 5′UTRs of mRNAs [38–40].

Based on previous lines of evidence and our findings, back-spliced exons and introns produce circRNAs co-transcriptionally. Subsequently, circRNAs accumulate in the most crucial regions for mRNAs processing and metabolism, i.e., cap and poly (A) tail regions, acting as miRNA sponges via the SLAS machinery by interacting with miR-3p or miR-5p. Consequently, circRNAs frequently get saturation by interacting with at most 5 miRNAs. These fine tuning processes reflect the regulatory networks between circRNAs, miRNAs and mRNAs (Figure 4C).

### Landscape of RNA length

We analyzed the length of each type of RNAs. Consequently, we found that the length for mRNA, circRNA and LncRNA varies greatly. For mRNAs, the median length is 2,446 nt, ranging from 80 to 43,816 nt. For circRNAs, the median length is 3,648 nt, ranging from 74 to 433,729 nt. For LncRNAs, the median length is 833 nt, ranging from 61 to 106,351 nt (Figure 4D, Supplementary Table S11, S12 and S13).

Amongst these RNAs, the shortest RNAs are 2 LncRNAs (mascRNA and lincRNA-NFIA-2) with 61 nt; whereas the longest RNA is a circRNA (circRNA-100723) with 433 729 nt (Table 1).

**Table 1:** Extremes of RNA length

RNA Type	Minimum Length (nt)	Gene Symbol	Maximum Length (nt)	Gene Symbol
mRNA	80	HOXA3 (NM-153632; NM-030661)	43,816	MUC16
circRNA	74	circRNA-104691	433,729	circRNA-100723
lncRNA	61	mascRNAlincRNA-NFIA-2	106,351	lincRNA-XIRP2-5

### miRNA microarray data

Based on miRNA microarray (GSE63492), we found there were 50 deregulated miRNAs passing student *t* test with a *P* value < 0.05. There were 31 up-regulated and 19 down-regulated miRNAs. The top 10 up-regulated and top 10 down-regulated miRNAs were listed in Supplementary Text S1.

We addressed the expression profiles of miRNAs in IDD using scoliotic NP as control [27] (GSE 19943), amongst which 10 miRNAs were upregulated and 67 miRNAs were downregulated. In addition to the difference of microarray platform (miRCURY™ LNA Array v.11.0 vs. miRCURY™ LNA Array v.18.0), we found that the expression profiles of miRNAs were largely different in NP samples in terms of normal, scoliotic and IDD. Collectively, these findings indicate that miRNAs play a role not only in the etiology of IDD, but in the underlying mechanisms of scoliosis.

### CircRNA expression profiles in IDD

Using the Arraystar Human Array analysis, we identified the expression profiles of 2,894 circRNAs (GSE67566). Amongst the 636 differentially expressed circRNAs, there were 354 up-regulated and 282 down-regulated. The top 10 up-regulated and top 10 down-regulated circRNAs were listed in Supplementary Text S2, which includes annotations as pertaining genes.

**Figure 5:**
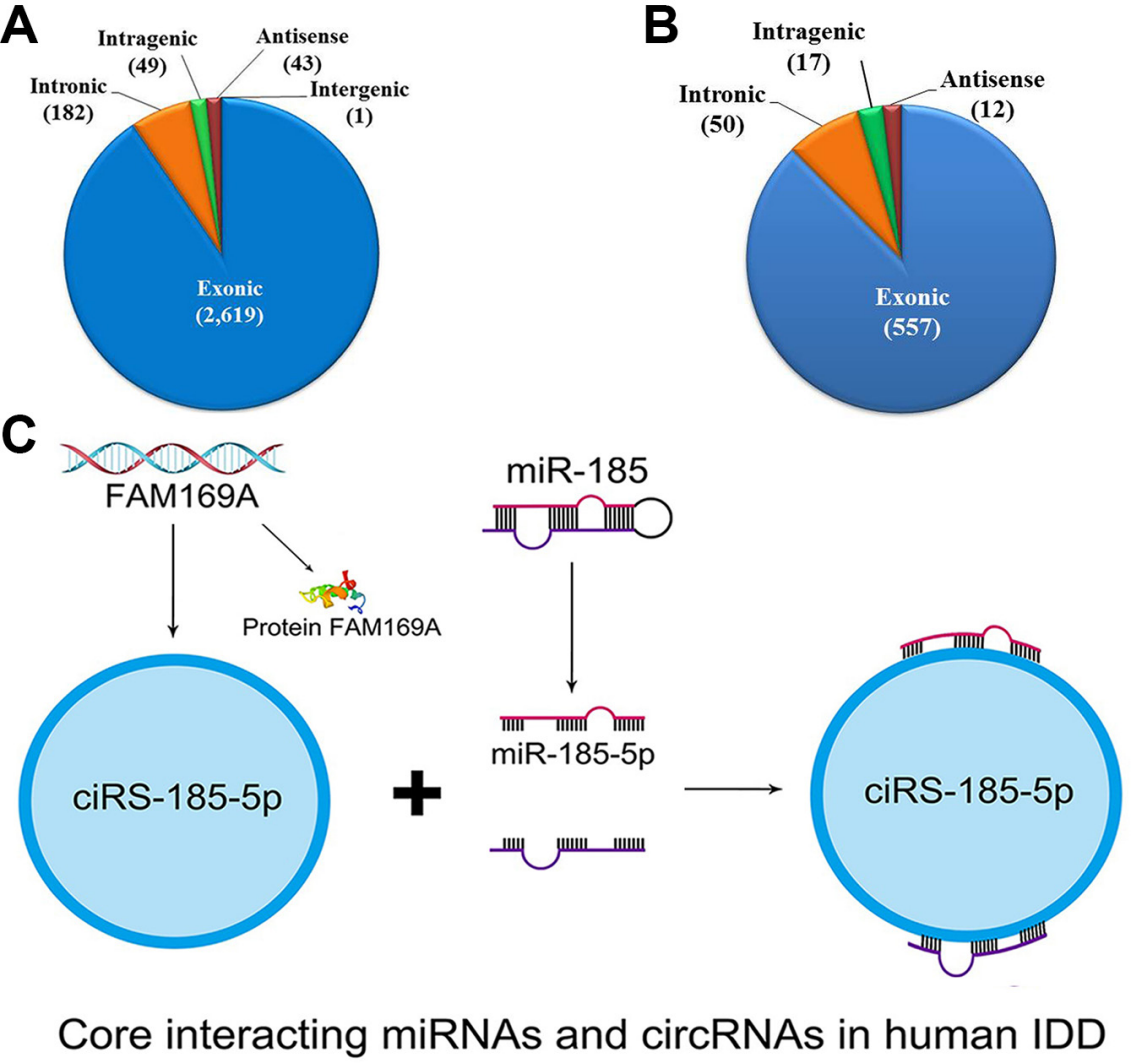
circRNAs and miRNAs in human intervertebral disc degeneration (**A**–**B**) Represent the constituent ratio of circRNAs and differentially expressed circRNAs. (**C**) indicates the core interacting miRNA, circRNA, and mRNA in human lumbar discs.

In general, the profiling consisted of 43 antisense (1.49%), 2619 exonic (90.50%), 1 intergenic (0.035%), 49 intragenic (1.69%), 182 intronic (6.29%) circRNAs (Figure 5A). The differentially expressed profiling consisted of 12 antisense (1.89%), 557 exonic (87.58%), 17 introgenic (2.67%), 50 intronic (7.86%) circRNAs. The distribution tread was similar with general profiling of circRNAs (Figure 5B).

### qRT-PCR validation

To validate the miRNAs and circRNAs microarray results, two miRNAs and two circRNAs were selected for qRT-PCR validation. The expression of hsa-miR-887-3p (*P* < 0.05) and has-circRNA-101852 (*P* < 0.05) was significantly increased in degenerative samples compared with control; whereas the expression of hsa-miR-125b-1-3p (*P* < 0.05) and has-circRNA-101645 (*P* < 0.05) were significantly decreased (Supplementary Figure S3). In summary, qRT-PCR validated the microarray outcome.

### Core interacting miRNAs and circRNAs in human IDD

Given that one miRNA could interact with a number of circRNAs and *vice versa*, we tried to narrow the scope and find valuable clues. We screened top 20 deregulated miRNAs in Supplementary Text S1 and top 20 deregulated circRNAs in Supplementary Text S2. Consequently, we found top 7 deregulated miRNAs may interact with 32 circRNAs via 84 potential binding sites; whereas 20 deregulated circRNAs may interact with 99 miRNAs via 143 potential binding sites. Notably, miR-328-5p could interact with 12 circRNAs via 49 binding sites; whereas circRNAs generally interact with 5 miRNAs (Supplementary PDF S2).

Amongst these linked miRNAs and circRNAs, we found circRNA-103890 (upregulated) generated by *FAM169A* (Gene ID: 26049, NM_015566*)* interacts with miR-185-5p (downregulated) (Figure 5C, Supplementary Figure S1). *FAM169A* contains 15 exons, coding its corresponding protein as soluble lamin-associated protein of 75 kDa (NP_056381, Uniprot identifiers: Q9Y6X4) [41, 42].

## DISCUSSION

The study is the first addressing mRNAs and ncRNAs in a type of human tissues rather than cultured cells. Furthermore, the expression profiling of RNAs sheds novel light on the understandings of not only the broadly influencing disease as lumbar IDD, but RNAs *per se*. These RNA hallmarks might reflect the true scenarios in human lumbar disc diseases and low back pain. Moreover, the novel vision might open a new page for RNA studies, based on RNA derivation from different Chromosomes. Strikingly, the length of mRNAs, circRNAs and ncRNAs varies to a great extent, the shortest of which is 2 lncRNAs (mascRNA and lincRNA-NFIA-2) with 61 nt. In contrast, the longest RNA is a circRNA (circRNA-100723) with 433,729 nt. These extreme RNAs might be the key to the treasure door of the truth on RNAs.

Traditionally, it has been reported that circRNAs derive from back-spliced exons [24, 25, 31, 32]. Surprisingly, we found nearly 10% circRNAs belong to intronic, antisense, intergenic rather than exonic types. Differentially expressed circRNAs remain the trend as shown in Figure 5B. The finding greatly expands our vision on circRNAs and splicing.

Accumulating evidence indicates that circRNAs expression has spatio-temporal features. Memczak et al. [32] identified 1,903 circRNAs in mouse tissues (brains, fetal head, differentiation-induced embryonic stem cells); 81 of which mapped to human circRNAs. Indeed, the brain is circRNAs abundant in human, mouse [31] and porcine [43]. The abundance of circRNAs is spatial and temporal depending, with the maximum in cortex at day 60 of gestation with 4,634 circRNAs [43]. As for H9 human embryonic stem cells, the abundance of circRNAs is 1,662 [26].

Bachmayr-Heyda et al. [44] compared the expression abundance of circRNAs in 13 human tissues. The most abundant tissue expressing circRNAs is brain and the lowest is muscle. In testis, Sry acts as miR-138 sponge [32]. We identified 2,894 circRNAs in human lumbar discs as a novel circRNA-abundant tissue.

Given that human brain, testis, stem cells and discs belong to immune privileged sites with FasL/Fas expression [45, 46], we putatively propose that circRNA expression might be tightly linked to immune privileged organs and tissues.

## MATERIALS AND METHODS

### Human NP sample collection

We collected human normal (cadaveric donors) and degenerative lumbar nucleus pulposus (NP) with patient demographics and IDD grading as we previously described [47]. Human disc bank was established with normal and degenerative samples and demographic records (Supplementary Figure S2).

### RNA isolation and quality control assay

Total RNA isolation and quality control assay were conducted as we previously describedas TRIspin method [47, 48].

### miRNA microarray analysis

The microarray platform for miRNAs was miRCURYTMLNA Array (v.18.0) (Exiqon, Vedbaek, Denmark). RNA labeling and array hybridization was performed according to Exiqon's manual. The miRCURY™ Hy3™/Hy5™ Power labeling kit (Exiqon, Vedbaek, Denmark) was used for miRNA labeling. The Hy3™-labeled samples were hybridized on the miRCURYTMLNA Array (v.18.0) (Exiqon) with subsequent hybridization using 12-Bay Hybridization Systems (Nimblegen Systems, Inc., Madison, WI, USA).

### circRNA array analysis

The sample preparation and microarray hybridization were performed based on the Arraystar's standard protocols. Labeled cRNAs were hybridized onto the Arraystar Human circRNA Array (8 × 15 K, Arraystar). Subsequently, Agilent Feature Extraction software (version 11.0.1.1) was used to analyze acquired array images. Quantile normalization and subsequent data processing were performed using the R software package. Hierarchical Clustering was performed to show the distinguishable circRNAs expression pattern among samples.

### LncRNA and mRNA microarray analysis

LncRNA and mRNA microarray analysis was conducted as we previously reported [47].

### Annotation for circRNA/miRNA interaction

Accumulating evidence indicates circRNAs play a crucial role in the delicate tuning of miRNA-mediated regulation of gene expression by sequestering corresponding miRNAs. The interacting machinery between circRNAs and miRNAs associated with diseases suggests that circular RNAs are important for disease regulation [49]. The circular RNA ciRS-7 contains various, tandem miRNA-7 binding sites, thereby acting as an endogenous miRNA “sponge” to adsorb, and hence quench, normal miRNA-7 functions [50]. ciRS-7 might serve as a crucial factor engaged in the functioning of neurons as well as a candidate in neurological disorders and tumor development on basis of several lines of evidence as follows: the widespread involvement of miR-7 as a key regulator of various cancer pathways; the suggested implications of miR-7 in Parkinson's disease by direct targeting of a-synuclein protein expression; direct targeting of the ubiquitin protein ligaseA (UBE2A) in Alzheimer's disease.

Therefore, circRNA/miRNA interaction was analyzed with Arraystar's home-made miRNA target prediction software based on TargetScan [51] & miRanda [52]. Differentially expressed circRNAs compared were annotated in details with the circRNA/miRNA interaction information.

### Quantitative Real-time PCR (qRT-PCR) assay

Candidate target genes were validated with highly reliable biotechniques such as qRT-PCR [53]. The primers were listed as Supplementary Text S3.

### Statistical analysis

Statistical analyses were performed using SPSS 19.0 software package. To analyze the expression difference of the particular lncRNAs or mRNAs in microarray and PCR analysis, student's *t-test* was applied to the study. Furthermore, the Benjamini-Hochberg FDR (the FDR cutoff was 0.05) was used for multiple-testing correction. The threshold of significance was set as *P-value* < 0.05.

## SUPPLEMENTARY MATERIALS FIGURES AND TABLES

































## References

[1] Gore M, Sadosky A, Stacey BR, Tai KS, Leslie D (2012). The burden of chronic low back pain: clinical comorbidities treatment patterns and health care costs in usual care settings. Spine (Phila Pa 1976).

[2] Battie MC, Videman T, Kaprio J, Gibbons LE, Gill K, Manninen H, Saarela J, Peltonen L (2009). The Twin Spine Study: contributions to a changing view of disc degeneration. Spine J.

[3] Battie MC, Videman T, Levalahti E, Gill K, Kaprio J (2008). Genetic and environmental effects on disc degeneration by phenotype and spinal level: a multivariate twin study. Spine (Phila Pa 1976).

[4] Song YQ, Karasugi T, Cheung KM, Chiba K, Ho DW, Miyake A, Kao PY, Sze KL, Yee A, Takahashi A, Kawaguchi Y, Mikami Y, Matsumoto M (2013). Lumbar disc degeneration is linked to a carbohydrate sulfotransferase 3 variant. J Clin Invest.

[5] Gruber HE, Hoelscher GL, Ingram JA, Bethea S, Zinchenko N, Hanley EN (2011). Variations in aggrecan localization and gene expression patterns characterize increasing stages of human intervertebral disk degeneration. Exp Mol Pathol.

[6] Gruber HE, Hoelscher GL, Ingram JA, Hanley EN (2012). Genome-wide analysis of pain- nerve- and neurotrophin -related gene expression in the degenerating human annulus. Mol Pain.

[7] Sun Z, Wang HQ, Liu ZH, Chang L, Chen YF, Zhang YZ, Zhang WL, Gao Y, Wan ZY, Che L, Liu X, Samartzis D, Luo ZJ (2013). Down-regulated CK8 expression in human intervertebral disc degeneration. Int J Med Sci.

[8] Sun Z, Guo YS, Yan SJ, Wan ZY, Gao B, Wang L, Liu ZH, Gao Y, Samartzis D, Lan LF, Wang HQ, Luo ZJ (2013). CK8 phosphorylation induced by compressive loads underlies the downregulation of CK8 in human disc degeneration by activating protein kinase C. Lab Invest.

[9] Bentley DR, Balasubramanian S, Swerdlow HP, Smith GP, Milton J, Brown CG, Hall KP, Evers DJ, Barnes CL, Bignell HR, Boutell JM, Bryant J, Carter RJ (2008). Accurate whole human genome sequencing using reversible terminator chemistry. Nature.

[10] Wheeler DA, Srinivasan M, Egholm M, Shen Y, Chen L, McGuire A, He W, Chen YJ, Makhijani V, Roth GT, Gomes X, Tartaro K, Niazi F (2008). The complete genome of an individual by massively parallel DNA sequencing. Nature.

[11] Brosnan CA, Voinnet O (2009). The long and the short of noncoding RNAs. Curr Opin Cell Biol.

[12] Twayana S, Legnini I, Cesana M, Cacchiarelli D, Morlando M, Bozzoni I (2013). Biogenesis and function of non-coding RNAs in muscle differentiation and in Duchenne muscular dystrophy. Biochem Soc Trans.

[13] Ahmed YL, Ficner R (2014). RNA synthesis and purification for structural studies. RNA Biol.

[14] Mishler DM, Gallivan JP (2014). A family of synthetic riboswitches adopts a kinetic trapping mechanism. Nucleic Acids Res.

[15] Costa FF (2010). Non-coding RNAs: Meet thy masters. Bioessays.

[16] Bartel DP (2009). MicroRNAs: target recognition and regulatory functions. Cell.

[17] Kim ED, Sung S (2012). Long noncoding RNA: unveiling hidden layer of gene regulatory networks. Trends Plant Sci.

[18] Li D, Wang Y, Zhang K, Jiao Z, Zhu X, Skogerboe G, Guo X, Chinnusamy V, Bi L, Huang Y, Dong S, Chen R, Kan Y (2011). Experimental RNomics and genomic comparative analysis reveal a large group of species-specific small non-message RNAs in the silkworm Bombyx mori. Nucleic Acids Res.

[19] Xue Z, Ye Q, Anson SR, Yang J, Xiao G, Kowbel D, Glass NL, Crosthwaite SK, Liu Y (2014). Transcriptional interference by antisense RNA is required for circadian clock function. Nature.

[20] Pauli A, Valen E, Lin MF, Garber M, Vastenhouw NL, Levin JZ, Fan L, Sandelin A, Rinn JL, Regev A, Schier AF (2012). Systematic identification of long noncoding RNAs expressed during zebrafish embryogenesis. Genome Res.

[21] Schaukowitch K, Joo JY, Liu X, Watts JK, Martinez C, Kim TK (2014). Enhancer RNA Facilitates NELF Release from Immediate Early Genes. Mol Cell.

[22] Kugel JF, Goodrich JA (2013). The regulation of mammalian mRNA transcription by lncRNAs: recent discoveries and current concepts. Epigenomics.

[23] Kapusta A, Feschotte C (2014). Volatile evolution of long noncoding RNA repertoires: mechanisms and biological implications. Trends Genet.

[24] Ashwal-Fluss R, Meyer M, Pamudurti NR, Ivanov A, Bartok O, Hanan M, Evantal N, Memczak S, Rajewsky N, Kadener S (2014). circRNA Biogenesis Competes with Pre-mRNA Splicing. Mol Cell.

[25] Vicens Q, Westhof E (2014). Biogenesis of Circular RNAs. Cell.

[26] Zhang XO, Wang HB, Zhang Y, Lu X, Chen LL, Yang L (2014). Complementary sequence-mediated exon circularization. Cell.

[27] Wang HQ, Yu XD, Liu ZH, Cheng X, Samartzis D, Jia LT, Wu SX, Huang J, Chen J, Luo ZJ (2011). Deregulated miR-155 promotes Fas-mediated apoptosis in human intervertebral disc degeneration by targeting FADD and caspase-3. J Pathol.

[28] Wan ZY, Song F, Sun Z, Chen YF, Zhang WL, Samartzis D, Ma CJ, Che L, Liu X, Ali MA, Wang HQ, Luo ZJ (2014). Aberrantly expressed long noncoding RNAs in human intervertebral disc degeneration: a microarray related study. Arthritis Res Ther.

[29] Roberts S, Menage J, Eisenstein SM (1993). The cartilage end-plate and intervertebral disc in scoliosis: calcification and other sequelae. J Orthop Res.

[30] Liu J, Mao K, Wang X, Guo W, Zhou L, Xu J, Liu Z, Mao K, Tang P (2015). Calcium Sulfate Hemihydrate/Mineralized Collagen for Bone Tissue Engineering: *In Vitro* Release and *In Vivo* Bone Regeneration Studies. J Biomater Tissue Eng.

[31] Hansen TB, Jensen TI, Clausen BH, Bramsen JB, Finsen B, Damgaard CK, Kjems J (2013). Natural RNA circles function as efficient microRNA sponges. Nature.

[32] Memczak S, Jens M, Elefsinioti A, Torti F, Krueger J, Rybak A, Maier L, Mackowiak SD, Gregersen LH, Munschauer M, Loewer A, Ziebold U, Landthaler M (2013). Circular RNAs are a large class of animal RNAs with regulatory potency. Nature.

[33] Glazar P, Papavasileiou P, Rajewsky N (2014). circBase: a database for circular RNAs. RNA.

[34] Dudekula DB, Panda AC, Grammatikakis I, De S, Abdelmohsen K, Gorospe M (2015). CircInteractome: a web tool for exploring circular RNAs and their interacting proteins and microRNAs. RNA Biol.

[35] Chang H, Lim J, Ha M, Kim VN (2014). TAIL-seq: genome-wide determination of poly(A) tail length and 3′ end modifications. Mol Cell.

[36] Subtelny AO, Eichhorn SW, Chen GR, Sive H, Bartel DP (2014). Poly(A)-tail profiling reveals an embryonic switch in translational control. Nature.

[37] Topisirovic I, Svitkin YV, Sonenberg N, Shatkin AJ (2011). Cap and cap-binding proteins in the control of gene expression. Wiley Interdiscip Rev RNA.

[38] Guo ZW, Xie C, Yang JR, Li JH, Yang JH, Zheng L (2015). MtiBase: a database for decoding microRNA target sites located within CDS and 5′UTR regions from CLIP-Seq and expression profile datasets. Database.

[39] Tay Y, Zhang J, Thomson AM, Lim B, Rigoutsos I (2008). MicroRNAs to Nanog Oct4 and Sox2 coding regions modulate embryonic stem cell differentiation. Nature.

[40] Lee I, Ajay SS, Yook JI, Kim HS, Hong SH, Kim NH, Dhanasekaran SM, Chinnaiyan AM, Athey BD (2009). New class of microRNA targets containing simultaneous 5′-UTR and 3′-UTR interaction sites. Genome Res.

[41] UniProtKB - Q9Y6X4 (F169A_HUMAN). http://www.uniprotorg/uniprot/Q9Y6X4.

[42] The Protein Model Portal. http://www.proteinmodelportalorg/query/uniprot/Q9Y6X4.

[43] Veno MT, Hansen TB, Veno ST, Clausen BH, Grebing M, Finsen B, Holm IE, Kjems J (2015). Spatio-temporal regulation of circular RNA expression during porcine embryonic brain development. Genome Biol.

[44] Bachmayr-Heyda A, Reiner AT, Auer K, Sukhbaatar N, Aust S, Bachleitner-Hofmann T, Mesteri I, Grunt TW, Zeillinger R, Pils D (2015). Correlation of circular RNA abundance with proliferation—exemplified with colorectal and ovarian cancer idiopathic lung fibrosis and normal human tissues. Sci Rep.

[45] Ma CJ, Liu X, Che L, Liu ZH, Samartzis D, Wang HQ (2015). Stem Cell Therapies for Intervertebral Disc Degeneration: Immune Privilege Reinforcement by Fas/FasL Regulating Machinery. Curr Stem Cell Res Ther.

[46] Liu ZH, Sun Z, Wang HQ, Ge J, Jiang TS, Chen YF, Ma Y, Wang C, Hu S, Samartzis D, Luo ZJ (2013). FasL expression on human nucleus pulposus cells contributes to the immune privilege of intervertebral disc by interacting with immunocytes. Int J Med Sci.

[47] Wang HQ, Samartzis D (2014). Clarifying the nomenclature of intervertebral disc degeneration and displacement: from bench to bedside. Int J Clin Exp Pathol.

[48] Reno C, Marchuk L, Sciore P, Frank CB, Hart DA (1997). Rapid isolation of total RNA from small samples of hypocellular dense connective tissues. Biotechniques.

[49] Ghosal S, Das S, Sen R, Basak P, Chakrabarti J (2013). Circ2Traits: a comprehensive database for circular RNA potentially associated with disease and traits. Front Genet.

[50] Lukiw WJ (2013). Circular RNA (circRNA) in Alzheimer's disease (AD). Front Genet.

[51] Enright AJ, John B, Gaul U, Tuschl T, Sander C, Marks DS (2003). MicroRNA targets in Drosophila. Genome Biol.

[52] Pasquinelli AE (2012). MicroRNAs and their targets: recognition regulation and an emerging reciprocal relationship. Nat Rev Genet.

[53] Yan B, Wang ZH, Guo JT (2012). The research strategies for probing the function of long noncoding RNAs. Genomics.

