# Supplementary material for: Landscape of RNAs in human lumbar disc degeneration
Source: Oncotarget. 2016 Aug 17;7(39):63166–76. doi: 10.18632/oncotarget.11334 (PMC5325354; doi:10.18632/oncotarget.11334)
Supplement: Supplementary file 1 [file oncotarget-07-63166-s001.pdf]

Landscape of RNAs in human lumbar disc degeneration

Supplementary Materials

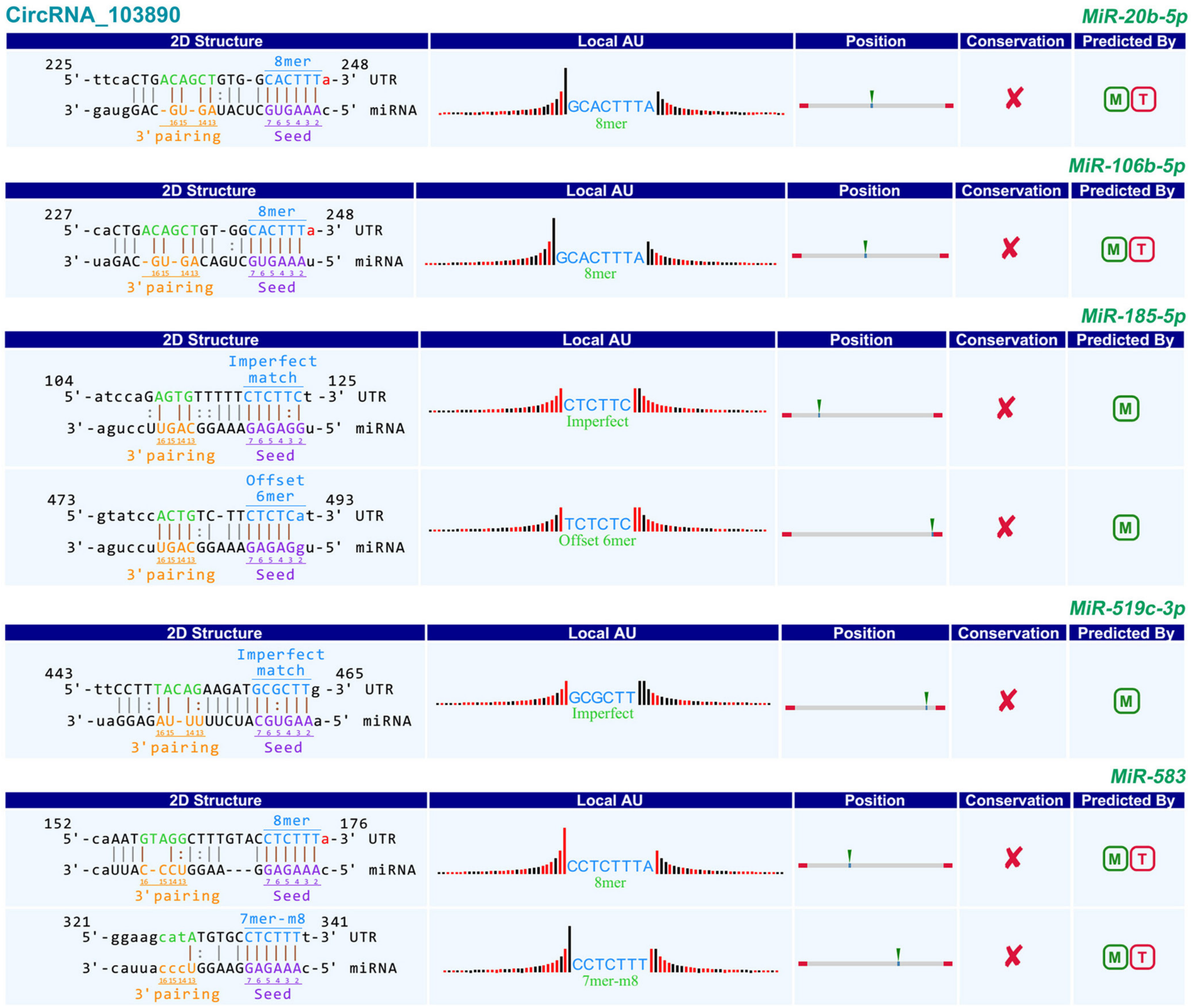

Supplementary Figure S1: Delineates binding sites of circRNA-103890 and 5 interactive miRNAs.

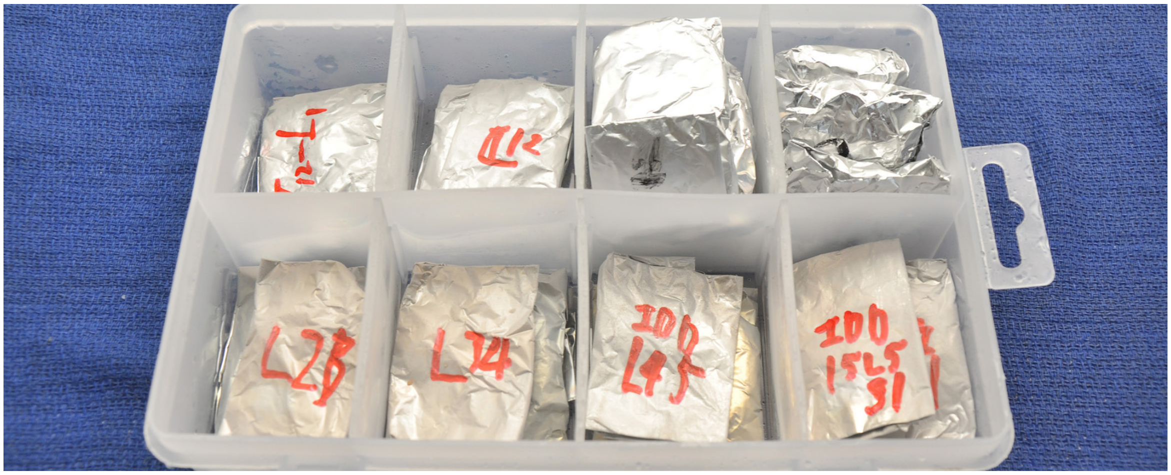

Supplementary Figure S2: Represents human intervertebral disc tissue banks established.

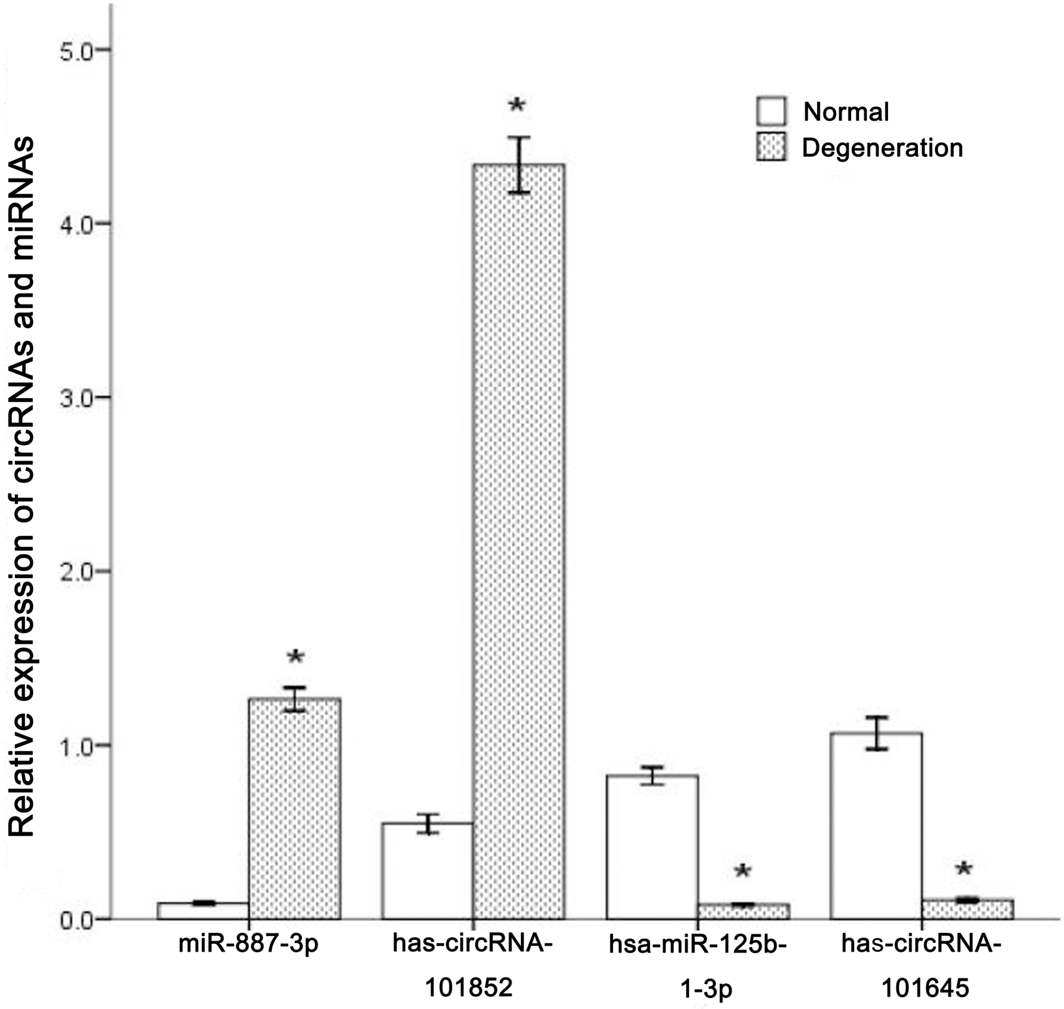

Supplementary Figure S3: Represents qRT-PCR validation of two circRNAs and two miRNAs. CircRNAs has-circRNA-101852, has-circRNA-101645 and miRNAs miR-887-3p, miR-125b-1-3p were chosen for the validation of the microarray outcome using qRT-PCR. The height of the columns in the figure represents relative expression of circRNAs and miRNAs; the bars represent standard deviation. Normal represents the normal disc group; Degeneration represents the degenerated disc group.*$P < 0.05$ versus the normal group.

Supplementary Table S1: Indicates mRNA expression profiling of GSE56081. See Supplementary_Table_S1

Supplementary Table S2: Includes miRNA expression profiling of GSE63492. See Supplementary_Table_S2

Supplementary Table S3: Includes lncRNA expression profiling of GSE56081. See Supplementary_Table_S3

Supplementary Table S4: Includes circRNA expression profiling of GSE67566. See Supplementary_Table_S4

Supplementary Table S5: Includes differentially expressed mRNAs in human intervertebral disc degeneration. See Supplementary_Table_S5

Supplementary Table S6: Indicates differentially expressed miRNAs in human intervertebral disc degeneration. See Supplementary_Table_S6

Supplementary Table S7: Indicates differentially expressed lncRNAs in human intervertebral disc degeneration. See Supplementary_Table_S7

Supplementary Table S8: Indicates differentially expressed circRNAs in human intervertebral disc degeneration. See Supplementary_Table_S8

Supplementary Table S9: Indicates all miRNAs (miRNA, miR-3p, miR-5p) interacting with circRNAs. See Supplementary_Table_S9

Supplementary Table S10: Indicates 20 circRNAs interacting with less than 5 circRNAs. See Supplementary_Table_S10

Supplementary Table S11: Indicates the length scope of mRNAs. See Supplementary_Table_S11

Supplementary Table S12: Indicates the length scope of circRNAs. See Supplementary_Table_S12

Supplementary Table S13: Indicates the length scope of lncRNAs. See Supplementary_Table_S13

Supplementary Texts 1: includes top 10 deregulated miRNAs in human intervertebral disc degeneration.

Table 1: Top 10 upregulated and downregulated miRNAs

Name	Fold change	*P* value	FDR
**Upregulated miRNAs**			
hsa-miR-3150a-3p	22.83876	0.003442	0.227991
hsa-miR-887-3p	14.49296	0.020397	0.343107
bkv-miR-B1-5p	12.22985	0.018847	0.343107
hsa-miR-516a-5p	9.405427	0.008099	0.293925
hsa-miR-431-5p	6.771374	0.019796	0.343107
hsa-miR-548at-5p	6.279139	0.043005	0.387107
kshv-miR-K12-2-5p	6.171413	0.010453	0.293925
hsa-miR-328-5p	5.951198	0.025355	0.346056
hcmv-miR-US33-5p	5.250919	0.011211	0.293925
hsa-miR-183-3p	5.214776	0.02082	0.343107
**Downregulated miRNAs**			
hsa-miR-125b-1-3p	0.068179	0.033463	0.37958
hsa-miR-1184	0.147331	0.008381	0.293925
ebv-miR-BART6-3p	0.153883	0.041563	0.387107
hsa-miR-3648	0.242209	0.011026	0.293925
hsa-miR-4769-5p	0.245242	2.49E-05	0.028008
hsa-miR-1273g-3p	0.24879	0.00221	0.227991
hsa-miR-4306	0.297107	0.002067	0.227991
hsa-miR-5100	0.3335	0.025394	0.346056
hsa-miR-185-5p	0.369146	0.003237	0.227991
hsa-miR-5002-5p	0.397484	0.033124	0.379573

FDR is calculated from Benjamini Hochberg false discovery rate.

Supplementary Texts 2: represents top 10 deregulated and classification circRNAs.

Table 2 Top 10 upregulated and downregulated circRNAs

Name	Fold change	*P* value	FDR	circRNA type	Gene Symbol
**Upregulated circRNAs**					
hsa_circRNA_101852	7.8935704	0	0	exonic	SNTB2
hsa_circRNA_101853	7.613375	0	0	exonic	SNTB2
hsa_circRNA_101139	7.5917155	0	0	exonic	ATP2A2
hsa_circRNA_103890	7.2722199	0	0	exonic	FAM169A
hsa_circRNA_400019	7.1555501	0	0	intronic	RPL27A
hsa_circRNA_102324	6.8569476	0	0	exonic	TMEM241
hsa_circRNA_104703	6.5818417	0	0	exonic	PTK2
hsa_circRNA_104600	6.4252965	0	0	exonic	VDAC3
hsa_circRNA_100604	6.3991222	0	0	exonic	DNA2
hsa_circRNA_100018	6.1182821	0	0	exonic	GNB1
**Downregulated circRNAs**					
hsa_circRNA_101645	9.8516471	0	0	exonic	SEMA4B
hsa_circRNA_104508	9.5513151	2E-11	0	exonic	PARP12
hsa_circRNA_102116	9.037889	0	0	exonic	ZNF652
hsa_circRNA_103838	8.322474	0	0	exonic	ARL15
hsa_circRNA_101557	8.273989	0	0	exonic	SPG21
hsa_circRNA_101709	8.2199898	0	0	exonic	GSPT1
hsa_circRNA_104630	8.2127228	0	0	exonic	RAB2A
hsa_circRNA_104019	7.8612435	2E-11	0	exonic	SFXN1
hsa_circRNA_101370	7.2577821	0	0	exonic	GPHN
hsa_circRNA_105031	7.0966142	0	0	exonic	MBNL3
**Classification of circRNAs**					
Exonic	circRNA arising from the exons of the linear transcript				
Intronic	circRNA arising from the introns of the linear transcript				
Antisense	circRNA whose gene locus overlap with the linear RNA, but transcribed from the opposite strand				
Intragenic	circRNA transcribed from same gene locus as the linear transcript, but not classified into “exonic” and “intronic”.				
Intergenic	circRNA located outside known gene locus				

FDR is calculated from Benjamini Hochberg false discovery rate.

Supplementary Texts 3: includes primer sequences for real-time PCR validation.

Table 3: miRNAs and circRNAs for qRT-PCR validation and the primer sequences

Gene	sense	Sequence 5′ →3′
hsa-miR-887-3p	GSP	GTGAACGGGCGCCATC
	R	GTGCGTGTCGTGGAGTCG
hsa-miR-125b-1-3p	GSP	GACGGGTTAGGCTCTTG
	R	GTGCGTGTCGTGGAGTCG
Has-circRNA-101852	F	TGGACCAGGATACTTGTTCA
	R	GTCCTCACTGCCACTAAAGC
Has-circRNA-101645	F	AGACGCAGAGAAGAAACAGCAG
	R	AGGAATGGCCGCTCTTCAGA
GAPDH	F	GGGAAACTGTGGCGTGAT
	R	GAGTGGGTGTCGCTGTTGA
U6	F	GCTTCGGCAGCACATATACTAAAAT
	R	CGCTTCACGAATTTGCGTGTCAT

Supplementary Texts 4: represents The Ratio of differently expressed RNAs within one single RNA type.

Table 4 The Ratio of differently expressed RNAs within one single RNA type

RNA Type	Total RNAs	Differentially expressed RNAs	Ratio
mRNAs	27547	2208	8.02%
miRNAs	2059	50	2.43%
LncRNAs	32660	3082	9.44%
circRNAs	5397	636	11.78%

Supplementary PDF S1: Includes all interacting miRNAs and circRNAs with their response elements details. See_ Supplementary_PDF_S1

Supplementary PDF S2: Includes screened interacting miRNAs and circRNAs. See_Supplementary_PDF_S2
