# Supplementary material for: Landscape of RNAs in human lumbar disc degeneration
Source: Oncotarget. 2016 Aug 17;7(39):63166–76. doi: 10.18632/oncotarget.11334 (PMC5325354; doi:10.18632/oncotarget.11334)
Supplement: Supplementary file 15 [file oncotarget-07-63166-s015.pdf]

Microarray analysis between Micro-RNA and Circ-RNA

(Based on Micro-RNA)

MiR-125b-1-3p

CircRNA_001040

2D Structure	Local AU	Position	Conservation	Predicted By
42 … 61 (Imperfect match)<br>5'-aggggCCA--GGCCTAGCCCGc -3' UTR<br>3'-ucgagGGUUCUCGGAUUGGGCa-5' miRNA<br>16 15 14 13 / 7 6 5 4 3 2<br>3'pairing Seed	AGCCCG<br>Imperfect		✘	M

CircRNA_101175

2D Structure	Local AU	Position	Conservation	Predicted By
54 … 72 (7mer-m8)<br>5'-tgcagCCAAGA---TAACCCGt-3' UTR<br>3'-ucgagGGUUCUCGGAUUGGGCa-5' miRNA<br>16 15 14 13 / 7 6 5 4 3 2<br>3'pairing Seed	TAACCCG<br>7mer-m8		✘	M T

MiR-183-3p

CircRNA_103942

2D Structure	Local AU	Position	Conservation	Predicted By
138 … 159 (7mer-m8)<br>5'-ccccaGCCCTTATTTAATTCAg-3' UTR<br>3'-aauacCGGGAAGCCAUUAAGUg-5' miRNA<br>16 15 14 13 / 7 6 5 4 3 2<br>3'pairing Seed	TAATTCA<br>7mer-m8		✘	M T

MiR-185-5p

CircRNA_000684

2D Structure	Local AU	Position	Conservation	Predicted By
497 … 518 (Imperfect match)<br>5'-ggtcaAGCTTCCTTTTTTTCCa -3' UTR<br>3'-aguccUUGACGGAAAGAGAGGu-5' miRNA<br>16 15 14 13 / 7 6 5 4 3 2<br>3'pairing Seed	TTTTCC<br>Imperfect		✘	M
892 … 913 (Imperfect match)<br>5'-gggtGAATTCCTTTTTTTTCCa -3' UTR<br>3'-agucCUUGACGGAAAGAGAGGu-5' miRNA<br>16 15 14 13 / 7 6 5 4 3 2<br>3'pairing Seed	TTTTCC<br>Imperfect		✘	M
931 … 954 (8mer)<br>5'-ccATGAACATGGAATATCTCTCCa-3' UTR<br>3'-agUCCUUG-A-CGGAAAGAGAGGu-5' miRNA<br>16 15 14 13 / 7 6 5 4 3 2<br>3'pairing Seed	TCTCTCCA<br>8mer		✘	M T
1439 … 1460 (Imperfect match)<br>5'-tttatcACATG-CTTTTTCTCCa -3' UTR<br>3'-aguccuUG-ACGGAAAGAGAGGu-5' miRNA<br>16 15 14 13 / 7 6 5 4 3 2<br>3'pairing Seed	TTCTCC<br>Imperfect		✘	M
2586 … 2608 (Imperfect match)<br>5'-gtAGGATTTGTATTGTTTCTCCc -3' UTR<br>3'-agUCCUUGACGGAA-AGAGAGGu-5' miRNA<br>16 15 14 13 / 7 6 5 4 3 2<br>3'pairing Seed	TTCTCC<br>Imperfect		✘	M

CircRNA_001175

2D Structure	Local AU	Position	Conservation	Predicted By
171 … 193 (8mer)<br>5'-acatgcgtccCCTCATCTCTCCa-3' UTR<br>3'-aguccuugacGGA-AAGAGAGGu-5' miRNA<br>16 15 14 13 / 7 6 5 4 3 2<br>3'pairing Seed	TCTCTCCA<br>8mer		✘	M T
250 … 273 (7mer-m8)<br>5'-tctcGATCTGGCCCAATCTCTCCt-3' UTR<br>3'-agucCUUGAC--GGAAAGAGAGGu-5' miRNA<br>16 15 14 13 / 7 6 5 4 3 2<br>3'pairing Seed	TCTCTCC<br>7mer-m8		✘	M T

CircRNA_100177

2D Structure	Local AU	Position	Conservation	Predicted By
167 … 187 (7mer-m8)<br>5'-gtactcACTG-CTGTCTCTCCt-3' UTR<br>3'-aguccuUGACGGAAAGAGAGGu-5' miRNA<br>16 15 14 13 / 7 6 5 4 3 2<br>3'pairing Seed	TCTCTCC<br>7mer-m8		✘	M T

CircRNA_100827

2D Structure	Local AU	Position	Conservation	Predicted By
85 … 110 (7mer-m8)<br>5'-ctGGAAACGTGTCCTGCATCTCTCCt-3' UTR<br>3'-agUCCUUG-ACGGAA---AGAGAGGu-5' miRNA<br>16 15 14 13 / 7 6 5 4 3 2<br>3'pairing Seed	TCTCTCC<br>7mer-m8		✘	M T

CircRNA_101183

2D Structure	Local AU	Position	Conservation	Predicted By
Offset 6mer<br>209 … 229<br>5'-attGGAGC-ATCTGTCTCTCTt-3' UTR |||:| :|| ||||||:<br>3'-aguCCUUGACGGAAAGAGAGGu-5' miRNA<br>16 15 14 13 … 7 6 5 4 3 2<br>3'pairing Seed	TCTCTC<br>Offset 6mer		✗	M
Offset 6mer<br>403 … 424<br>5'-tgAGTTGCTGTCATTCTCTCTg-3' UTR || :|||:| |||||||:<br>3'-aguCCUUGACGGAAAGAGAGGu-5' miRNA<br>16 15 14 13 … 7 6 5 4 3 2<br>3'pairing Seed	TCTCTC<br>Offset 6mer		✗	M

CircRNA_101995

2D Structure	Local AU	Position	Conservation	Predicted By
8mer<br>407 … 428<br>5'-actggctgaGCCTGTCTCTCCa-3' UTR |||| ||||||| 3'-aguccuugaCGGAAAGAGAGGu-5' miRNA<br>16 15 14 13 … 7 6 5 4 3 2<br>3'pairing Seed	TCTCTCCA<br>8mer		✗	M T
7mer-m8<br>587 … 610<br>5'-atgtGACAGTGCCCATTCTCTCCg-3' UTR ||	|| || |||||||| 3'-agucCU-UGAC-GGAAAGAGAGGu-5' miRNA<br>16 15 14 13 … 7 6 5 4 3 2<br>3'pairing Seed	TCTCTCC<br>7mer-m8		✗	M T
Imperfect match<br>999 … 1020<br>5'-tacctGGCTGACATTCTCTTCt-3' UTR<br>::|||	|||||:| 3'-aguccUUGACGGAAAGAGAGGu-5' miRNA<br>16 15 14 13 … 7 6 5 4 3 2<br>3'pairing Seed	CTCTTC<br>Imperfect		✗	M

CircRNA_102122

2D Structure	Local AU	Position	Conservation	Predicted By
Imperfect match<br>351 … 373<br>5'-ggAGAAACCTCCATTTTTCTCCg-3' UTR || || ||	|||:||||| 3'-agUCCUU-GACGGAAAGAGAGGu-5' miRNA<br>16 15 14 13 … 7 6 5 4 3 2<br>3'pairing Seed	TTCTCC<br>Imperfect		✗	M
7mer-m8<br>406 … 427<br>5'-accaGCGCTGGCTTTCTCTCCc-3' UTR | :||| |||||||||| 3'-agucCUUGACGGAAAGAGAGGu-5' miRNA<br>16 15 14 13 … 7 6 5 4 3 2<br>3'pairing Seed	TCTCTCC<br>7mer-m8		✗	M T

CircRNA_103890

2D Structure	Local AU	Position	Conservation	Predicted By
Imperfect match<br>104 … 125<br>5'-atccaGAGTGTTTTTCTCTTCt-3' UTR<br>:| ||::|||||||:| 3'-aguccUUGACGGAAAGAGAGGu-5' miRNA<br>16 15 14 13 … 7 6 5 4 3 2<br>3'pairing Seed	CTCTTC<br>Imperfect		✗	M
Offset 6mer<br>473 … 493<br>5'-gtatccACTGTC-TTCTCTCat-3' UTR ||||:| |||||| 3'-aguccuUGACGGAAAGAGAGgu-5' miRNA<br>16 15 14 13 … 7 6 5 4 3 2<br>3'pairing Seed	TCTCTC<br>Offset 6mer		✗	M

CircRNA_104242

2D Structure	Local AU	Position	Conservation	Predicted By
8mer<br>129 … 151<br>5'-acatgcgtccCCTCATCTCTCCa-3' UTR ||| ||||||| 3'-aguccuugacGGA-AAGAGAGGu-5' miRNA<br>16 15 14 13 … 7 6 5 4 3 2<br>3'pairing Seed	TCTCTCCA<br>8mer		✗	M T
7mer-m8<br>208 … 231<br>5'-tctcGATCTGGCCCAATCTCTCCt-3' UTR || ||| || ||||||| 3'-agucCUUGAC--GGAAAGAGAGGu-5' miRNA<br>16 15 14 13 … 7 6 5 4 3 2<br>3'pairing Seed	TCTCTCC<br>7mer-m8		✗	M T

CircRNA_104243

2D Structure	Local AU	Position	Conservation	Predicted By
8mer<br>129 … 151<br>5'-acatgcgtccCCTCATCTCTCCa-3' UTR ||| ||||||| 3'-aguccuugacGGA-AAGAGAGGu-5' miRNA<br>16 15 14 13 … 7 6 5 4 3 2<br>3'pairing Seed	TCTCTCCA<br>8mer		✗	M T
7mer-m8<br>208 … 231<br>5'-tctcGATCTGGCCCAATCTCTCCt-3' UTR || ||| || ||||||| 3'-agucCUUGAC--GGAAAGAGAGGu-5' miRNA<br>16 15 14 13 … 7 6 5 4 3 2<br>3'pairing Seed	TCTCTCC<br>7mer-m8		✗	M T

CircRNA_104414

<table>
<tr><th>2D Structure</th><th>Local AU</th><th>Position</th><th>Conservation</th><th>Predicted By</th></tr>
<tr><td>Offset 6mer
70 91
5'-ggAGAAGCTGACTTTCTCTCTg-3' UTR
|| |:||| ||||||||:
3'-agUCCUUGACGGAAAGAGAGGu-5' miRNA
16 15 14 13 7 6 5 4 3 2
3'pairing Seed</td><td>TCTCTC
Offset 6mer</td><td></td><td>✗</td><td>M</td></tr>
</table>

MiR-328-5p

CircRNA_000941

<table>
<tr><th>2D Structure</th><th>Local AU</th><th>Position</th><th>Conservation</th><th>Predicted By</th></tr>
<tr><td>Imperfect match
1052 1078
5'-ttgTGATCCGCCTGCCTCGGCCTCCCa -3' UTR
||| || ||| ||| |||:|||
3'-gggACUCGG-GGA-GGA--CGGGGGGg-5' miRNA
16 15 14 13 7 6 5 4 3 2
3'pairing Seed</td><td>CCTCCC
Imperfect</td><td></td><td>✗</td><td>M</td></tr>
<tr><td>Imperfect match
3176 3202
5'-aagTGATTCTCCTGCCTTAGCCTCCCa -3' UTR
||| |:||| ||| |||:|||
3'-gggACU-CGGGGA-GGA--CGGGGGGg-5' miRNA
16 15 14 13 7 6 5 4 3 2
3'pairing Seed</td><td>CCTCCC
Imperfect</td><td></td><td>✗</td><td>M</td></tr>
<tr><td>7mer-m8
3356 3380
5'-gcCTGAGCCACTGGCCTGCCCCCCc-3' UTR
||||||| || |||||||||
3'-ggGACUCGGGGA--GGACGGGGGGg-5' miRNA
16 15 14 13 7 6 5 4 3 2
3'pairing Seed</td><td>GCCCCCC
7mer-m8</td><td></td><td>✗</td><td>M T</td></tr>
<tr><td>Imperfect match
5815 5840
5'-aagTGATCCACCCACCTTGCCCTCCc -3' UTR
||| | ||| || |||||:||
3'-gggACUCG--GGGAGG-ACGGGGGGg-5' miRNA
16 15 14 13 7 6 5 4 3 2
3'pairing Seed</td><td>CCCTCC
Imperfect</td><td></td><td>✗</td><td>M</td></tr>
<tr><td>Imperfect match
8823 8849
5'-tagTGATCCTCCTGCCTCAGCCTCCCa -3' UTR
||| || ||| ||| |||:|||
3'-gggACUCGG-GGA-GGA--CGGGGGGg-5' miRNA
16 15 14 13 7 6 5 4 3 2
3'pairing Seed</td><td>CCTCCC
Imperfect</td><td></td><td>✗</td><td>M</td></tr>
<tr><td>Offset 6mer
13254 13280
5'-aagaGATCCTCTCGCCTCAGCCCCCTg-3' UTR
|| ||:|| ||| ||||||:
3'-gggaCUCGGGGA--GGA--CGGGGGGg-5' miRNA
16 15 14 13 7 6 5 4 3 2
3'pairing Seed</td><td>GCCCCC
Offset 6mer</td><td></td><td>✗</td><td>M</td></tr>
<tr><td>Imperfect match
13761 13783
5'-ctcactGCAACTTCTGCCTCCCa -3' UTR
|| ||:|||||:|||
3'-gggacuCGGGGAGGACGGGGGGg-5' miRNA
16 15 14 13 7 6 5 4 3 2
3'pairing Seed</td><td>CCTCCC
Imperfect</td><td></td><td>✗</td><td>M</td></tr>
<tr><td>Imperfect match
13932 13953
5'-atCTGCCCACCT-CTGCCTCCCa -3' UTR
||| | ||| |||||:|||
3'-ggGACUCGGGGAGGACGGGGGGg-5' miRNA
16 15 14 13 7 6 5 4 3 2
3'pairing Seed</td><td>CCTCCC
Imperfect</td><td></td><td>✗</td><td>M</td></tr>
<tr><td>Imperfect match
16423 16445
5'-cattttcCTGCTTCAGCCTCCCg -3' UTR
|: ||:| |||:|||
3'-gggacucGGGGAGGACGGGGGGg-5' miRNA
16 15 14 13 7 6 5 4 3 2
3'pairing Seed</td><td>CCTCCC
Imperfect</td><td></td><td>✗</td><td>M</td></tr>
<tr><td>Imperfect match
16558 16579
5'-atCTGCCCGCCT-CGGCCTCCCa -3' UTR
||| | ||| | |||:|||
3'-ggGACUCGGGGAGGACGGGGGGg-5' miRNA
16 15 14 13 7 6 5 4 3 2
3'pairing Seed</td><td>CCTCCC
Imperfect</td><td></td><td>✗</td><td>M</td></tr>
<tr><td>7mer-m8
16998 17018
5'-taCTCAG--ATTCTTGCCCCCCc-3' UTR
|| || :||:|||||||
3'-ggGACUCGGGGAGGACGGGGGGg-5' miRNA
16 15 14 13 7 6 5 4 3 2
3'pairing Seed</td><td>GCCCCCC
7mer-m8</td><td></td><td>✗</td><td>M T</td></tr>
</table>

CircRNA_001072

<table>
<tr><th>2D Structure</th><th>Local AU</th><th>Position</th><th>Conservation</th><th>Predicted By</th></tr>
<tr><td>Imperfect match
16 38
5'-gtCTGACCGCAACCTGCCCTTCg -3' UTR
|||| | | |||||||::|
3'-ggGACUCGGGGAGGACGGGGGGg-5' miRNA
16 15 14 13 7 6 5 4 3 2
3'pairing Seed</td><td>CCCTTC
Imperfect</td><td></td><td>✗</td><td>M</td></tr>
</table>

CircRNA_001396

<table>
<tr><th>2D Structure</th><th>Local AU</th><th>Position</th><th>Conservation</th><th>Predicted By</th></tr>
<tr><td>7mer-m8
53 75
5'-gcgcttgaaCCTGGAGCCCCCCg-3' UTR
||| |||||||
3'-gggacucggGGAGGACGGGGGGg-5' miRNA
16 15 14 13 7 6 5 4 3 2
3'pairing Seed</td><td>GCCCCCC
7mer-m8</td><td></td><td>✗</td><td>M T</td></tr>
<tr><td>Imperfect match
1227 1252
5'-ctCAGAGGTCCCCATCCTGCCTTCCc -3' UTR
| ||| |||| |||||||::||
3'-ggGACUC--GGGG-AGGACGGGGGGg-5' miRNA
16 15 14 13 7 6 5 4 3 2
3'pairing Seed</td><td>CCTTCC
Imperfect</td><td></td><td>✗</td><td>M</td></tr>
<tr><td>8mer
2996 3018
5'-agatgccagtgaaacGCCCCCCa-3' UTR
|||||||
3'-gggacucggggaggaCGGGGGGg-5' miRNA
16 15 14 13 7 6 5 4 3 2
3'pairing Seed</td><td>GCCCCCCA
8mer</td><td></td><td>✗</td><td>M T</td></tr>
<tr><td>Imperfect match
3475 3496
5'-cccgccGCGCCT-TTGCCCTCCg -3' UTR
|| ||| :||||||:||
3'-gggacuCGGGGAGGACGGGGGGg-5' miRNA
16 15 14 13 7 6 5 4 3 2
3'pairing Seed</td><td>CCCTCC
Imperfect</td><td></td><td>✗</td><td>M</td></tr>
<tr><td>Imperfect match
3638 3660
5'-acCGGAGCCCGCCCCGCCCCTCc -3' UTR
| ||||||| || |||||:|
3'-ggGACUCGGGGAGGACGGGGGGg-5' miRNA
16 15 14 13 7 6 5 4 3 2
3'pairing Seed</td><td>CCCCTC
Imperfect</td><td></td><td>✗</td><td>M</td></tr>
<tr><td>8mer
3756 3778
5'-ctCTGATCCCGCGCGGCCCCCCa-3' UTR
|||| ||| | |||||||
3'-ggGACUCGGGGAGGACGGGGGGg-5' miRNA
16 15 14 13 7 6 5 4 3 2
3'pairing Seed</td><td>GCCCCCCA
8mer</td><td></td><td>✗</td><td>M T</td></tr>
<tr><td>Imperfect match
4470 4492
5'-acacaacaCCCGCCAGTCCCCCa -3' UTR
||| || |:|||||
3'-gggacucgGGGAGGACGGGGGGg-5' miRNA
16 15 14 13 7 6 5 4 3 2
3'pairing Seed</td><td>TCCCCC
Imperfect</td><td></td><td>✗</td><td>M</td></tr>
<tr><td>Imperfect match
4523 4543
5'-ggCTGA--TTTTCTTGCCCCTCc -3' UTR
|||| :::||:||||||:|
3'-ggGACUCGGGGAGGACGGGGGGg-5' miRNA
16 15 14 13 7 6 5 4 3 2
3'pairing Seed</td><td>CCCCTC
Imperfect</td><td></td><td>✗</td><td>M</td></tr>
<tr><td>Imperfect match
4533 4555
5'-cttgcccCTCCACCAGCCTCCCa -3' UTR
|:|| || |||:|||
3'-gggacucGGGGAGGACGGGGGGg-5' miRNA
16 15 14 13 7 6 5 4 3 2
3'pairing Seed</td><td>CCTCCC
Imperfect</td><td></td><td>✗</td><td>M</td></tr>
<tr><td>Imperfect match
5355 5378
5'-gttcacGCCATTCTCAGCCTCCCa -3' UTR
||| :|| | |||:|||
3'-gggacuCGGGGAG-GACGGGGGGg-5' miRNA
16 15 14 13 7 6 5 4 3 2
3'pairing Seed</td><td>CCTCCC
Imperfect</td><td></td><td>✗</td><td>M</td></tr>
<tr><td>Imperfect match
5486 5512
5'-tcaTGATCCGCCCACCTCGGCCTCCCa -3' UTR
||| | ||| ||| |||:|||
3'-gggACUCG--GGGAGGA--CGGGGGGg-5' miRNA
16 15 14 13 7 6 5 4 3 2
3'pairing Seed</td><td>CCTCCC
Imperfect</td><td></td><td>✗</td><td>M</td></tr>
<tr><td>Imperfect match
6015 6036
5'-tcactgcaaCCTCC-GCCTCCCa -3' UTR
||||| |||:|||
3'-gggacucggGGAGGACGGGGGGg-5' miRNA
16 15 14 13 7 6 5 4 3 2
3'pairing Seed</td><td>CCTCCC
Imperfect</td><td></td><td>✗</td><td>M</td></tr>
<tr><td>Imperfect match
6229 6251
5'-cagTGCGCCCGGCCCGCCTCCCa -3' UTR
|| |||| || |||:|||
3'-gggACUCGGGGAGGACGGGGGGg-5' miRNA
16 15 14 13 7 6 5 4 3 2
3'pairing Seed</td><td>CCTCCC
Imperfect</td><td></td><td>✗</td><td>M</td></tr>
</table>

CircRNA_100188

2D Structure	Local AU	Position	Conservation	Predicted By
Imperfect match<br>686 710<br>5'-cccgcAGCCCTTCCCGGGCCCCTCa -3' UTR<br>3'-gggacUCGGGGAGG--ACGGGGGGg-5' miRNA<br>3'pairing Seed	CCCCTC<br>Imperfect		✘	M
8mer<br>752 777<br>5'-gcagaAGCCCCCCAAAAGGCCCCCCa-3' UTR<br>3'-gggacUCGGGGAG---GACGGGGGGg-5' miRNA<br>3'pairing Seed	GCCCCCCA<br>8mer		✘	M T
8mer<br>1718 1742<br>5'-tgCAGAGCAGCAGGTCAGCCCCCCa-3' UTR<br>3'-ggGACUCG--GGGAGGACGGGGGGg-5' miRNA<br>3'pairing Seed	GCCCCCCA<br>8mer		✘	M T
Imperfect match<br>1829 1851<br>5'-cacccAGCCCCTCCTGCCCCTgt -3' UTR<br>3'-gggacUCGGGGAGGACGGGGGgg-5' miRNA<br>3'pairing Seed	CCCCTG<br>Imperfect		✘	M
7mer-m8<br>2099 2122<br>5'-gcacccGCCCAGTACCGCCCCCCc-3' UTR<br>3'-gggacuCGGG-GAGGACGGGGGGg-5' miRNA<br>3'pairing Seed	GCCCCCC<br>7mer-m8		✘	M T
Imperfect match<br>3006 3028<br>5'-ccaTCACCCCCACCTGCCCCACa -3' UTR<br>3'-gggACUCGGGGAGGACGGGGGGg-5' miRNA<br>3'pairing Seed	CCCCAC<br>Imperfect		✘	M
Offset 6mer<br>3125 3147<br>5'-caCAGAGCCAGAGCCGCCCCCTa-3' UTR<br>3'-ggGACUCGGGGAGGACGGGGGGg-5' miRNA<br>3'pairing Seed	GCCCCC<br>Offset 6mer		✘	M
Offset 6mer<br>3923 3945<br>5'-tgagaAGCCATACCTGCCCCCac-3' UTR<br>3'-gggacUCGGGGAGGACGGGGGgg-5' miRNA<br>3'pairing Seed	GCCCCC<br>Offset 6mer		✘	M
Imperfect match<br>4378 4399<br>5'-ggCT-ACCCCCTGTGGCTCCCCc -3' UTR<br>3'-ggGACUCGGGGAGGACGGGGGGg-5' miRNA<br>3'pairing Seed	CTCCCC<br>Imperfect		✘	M
Imperfect match<br>5667 5689<br>5'-gaggaGGATGCTCCTGCCTCCCa -3' UTR<br>3'-gggacUCGGGGAGGACGGGGGGg-5' miRNA<br>3'pairing Seed	CCTCCC<br>Imperfect		✘	M

CircRNA_100433

2D Structure	Local AU	Position	Conservation	Predicted By
8mer<br>88 114<br>5'-gaaaGAGCCTTGCCACGAGGCCCCCCa-3' UTR<br>3'-gggaCUCGGGGAGG----ACGGGGGGg-5' miRNA<br>3'pairing Seed	GCCCCCCA<br>8mer		✘	M T
7mer-A1<br>678 700<br>5'-tcaTGATCCCAGCCTTCCCCCCa-3' UTR<br>3'-gggACUCGGGGAGGACGGGGGGg-5' miRNA<br>3'pairing Seed	TCCCCCCA<br>7mer-A1		✘	M T

CircRNA_100508

2D Structure	Local AU	Position	Conservation	Predicted By
Imperfect match<br>174 198<br>5'-ctacGACGCCTCGCCCTGCTCCCCg -3' UTR<br>3'-gggaCU-CGGGG-AGGACGGGGGGg-5' miRNA<br>3'pairing Seed	CTCCCC<br>Imperfect		✘	M
Offset 6mer<br>265 290<br>5'-gaCTGGGCCTTTGTGCCCGCCCCCTg-3' UTR<br>3'-ggGACUCGGGGA---GGACGGGGGGg-5' miRNA<br>3'pairing Seed	GCCCCC<br>Offset 6mer		✘	M

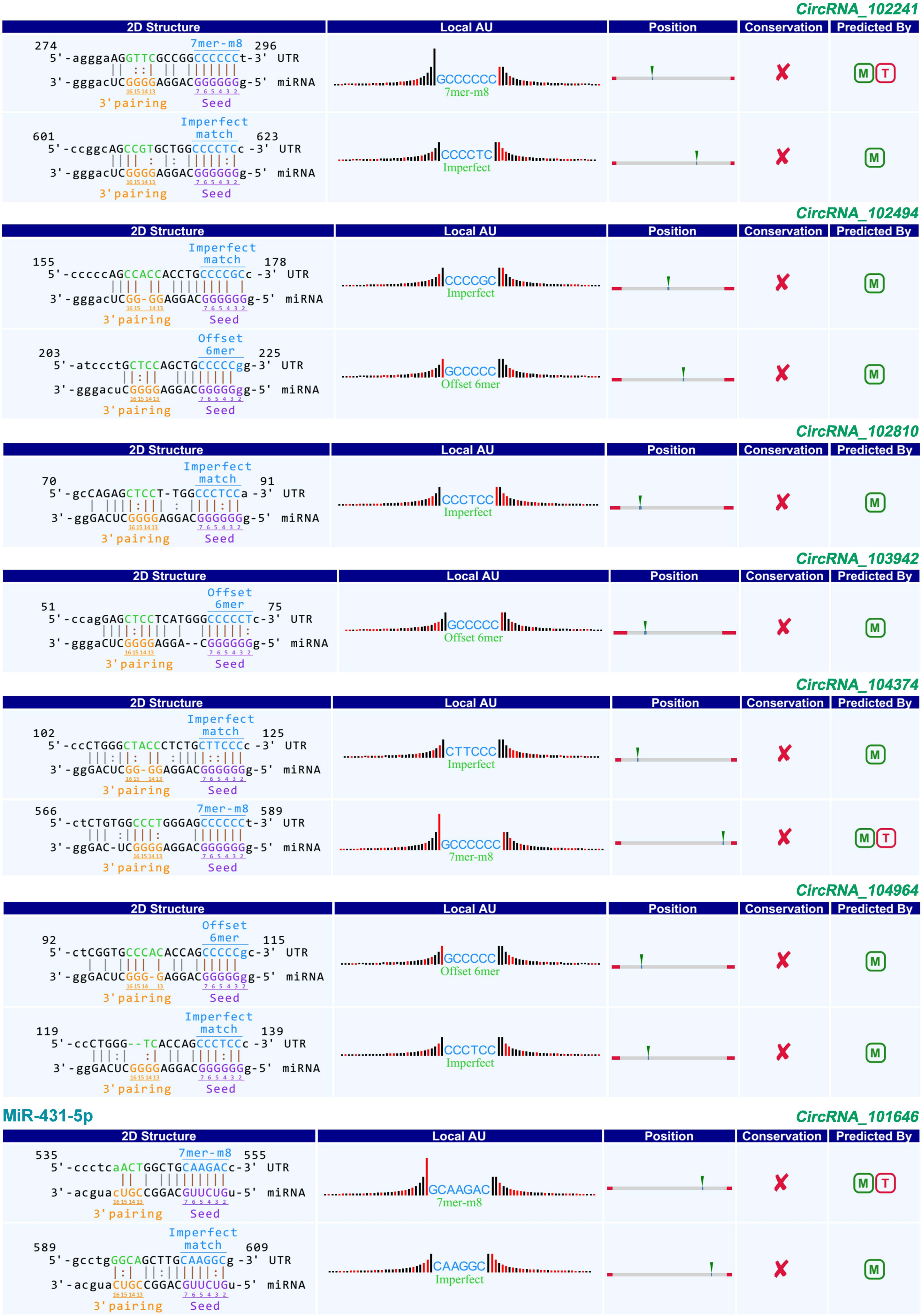

CircRNA_102241
2D Structure
Local AU
Position
Conservation
Predicted By
274
7mer-m8
296
5'-agggaAGGTTCGCCGGCCCCCCt-3' UTR
3'-gggacUCGGGGAGGACGGGGGGg-5' miRNA
3'pairing
Seed
GCCCCCC
7mer-m8
M
T
601
Imperfect match
623
5'-ccggcAGCCGTGCTGGCCCCTCc-3' UTR
3'-gggacUCGGGGAGGACGGGGGGg-5' miRNA
3'pairing
Seed
CCCCTC
Imperfect
M
CircRNA_102494
155
Imperfect match
178
5'-cccccAGCCACCACCTGCCCCGCc-3' UTR
3'-gggacUCGG-GGAGGACGGGGGGg-5' miRNA
3'pairing
Seed
CCCCGC
Imperfect
M
203
Offset 6mer
225
5'-atccctGCTCCAGCTGCCCCCgg-3' UTR
3'-gggacuCGGGGAGGACGGGGGgg-5' miRNA
3'pairing
Seed
GCCCCC
Offset 6mer
M
CircRNA_102810
70
Imperfect match
91
5'-gcCAGAGCTCCT-TGGCCCTCCa-3' UTR
3'-ggGACUCGGGGAGGACGGGGGGg-5' miRNA
3'pairing
Seed
CCCTCC
Imperfect
M
CircRNA_103942
51
Offset 6mer
75
5'-ccagGAGCTCCTCATGGGCCCCCTc-3' UTR
3'-gggaCUCGGGGAGGA--CGGGGGGg-5' miRNA
3'pairing
Seed
GCCCCC
Offset 6mer
M
CircRNA_104374
102
Imperfect match
125
5'-ccCTGGGCTACCCTCTGCTTCCCc-3' UTR
3'-ggGACUCGG-GGAGGACGGGGGGg-5' miRNA
3'pairing
Seed
CTTCCC
Imperfect
M
566
7mer-m8
589
5'-ctCTGTGGCCCTGGGAGCCCCCCt-3' UTR
3'-ggGAC-UCGGGGAGGACGGGGGGg-5' miRNA
3'pairing
Seed
GCCCCCC
7mer-m8
M
T
CircRNA_104964
92
Offset 6mer
115
5'-ctCGGTGCCCACACCAGCCCCCgc-3' UTR
3'-ggGACUCGGG-GAGGACGGGGGgg-5' miRNA
3'pairing
Seed
GCCCCC
Offset 6mer
M
119
Imperfect match
139
5'-ccCTGGG--TCACCAGCCCTCCc-3' UTR
3'-ggGACUCGGGGAGGACGGGGGGg-5' miRNA
3'pairing
Seed
CCCTCC
Imperfect
M
MiR-431-5p
CircRNA_101646
535
7mer-m8
555
5'-ccctcaACTGGCTGCAAGACc-3' UTR
3'-acguacUGCCGGACGUUCUGu-5' miRNA
3'pairing
Seed
GCAAGAC
7mer-m8
M
T
589
Imperfect match
609
5'-gcctgGGCAGCTTGCAAGGCg-3' UTR
3'-acguaCUGCCGGACGUUCUGu-5' miRNA
3'pairing
Seed
CAAGGC
Imperfect
M

CircRNA_100714

2D Structure	Local AU	Position	Conservation	Predicted By
238 7mer-m8 258<br>5'-tccagactctagTGCAAGACc-3' UTR<br>3'-acguacugccggACGUUCUGu-5' miRNA<br>16 15 14 13 7 6 5 4 3 2<br>3'pairing Seed	GCAAGAC<br>7mer-m8		✗	M T
615 8mer 635<br>5'-caataataaatgaGCAAGACa-3' UTR<br>3'-acguacugccggaCGUUCUGu-5' miRNA<br>16 15 14 13 7 6 5 4 3 2<br>3'pairing Seed	GCAAGACA<br>8mer		✗	M T
Imperfect match<br>1434 1454<br>5'-tgCATGACAGCCTGCATGACt -3' UTR<br>3'-acGUACUGCCGGACGUUCUGu-5' miRNA<br>16 15 14 13 7 6 5 4 3 2<br>3'pairing Seed	CATGAC<br>Imperfect		✗	M

MiR-516a-5p

CircRNA_101178

2D Structure	Local AU	Position	Conservation	Predicted By
225 8mer 248<br>5'-agAAGTCCACATTCCCCTCGAGAa-3' UTR<br>3'-cuUUCACGAAGAA-AGGAGCUCUu-5' miRNA<br>16 15 14 13 7 6 5 4 3 2<br>3'pairing Seed	CTCGAGAA<br>8mer		✗	M T

CircRNA_102509

2D Structure	Local AU	Position	Conservation	Predicted By
34 7mer-m8 56<br>5'-atcgtccaataccaCCTCGAGAt-3' UTR<br>3'-cuuucacgaagaaaGGAGCUCUu-5' miRNA<br>16 15 14 13 7 6 5 4 3 2<br>3'pairing Seed	CTCGAGA<br>7mer-m8		✗	M T
Imperfect match<br>127 149<br>5'-cacAGTG-TTCTTTGCCTCAAGAc -3' UTR<br>3'-cuuUCACGAAGAAA-GGAGCUCUu-5' miRNA<br>16 15 14 13 7 6 5 4 3 2<br>3'pairing Seed	TCAAGA<br>Imperfect		✗	M

CircRNA_104039

2D Structure	Local AU	Position	Conservation	Predicted By
246 7mer-m8 269<br>5'-caGATTGTCTCTGGATCTCGAGAt-3' UTR<br>3'-cuUUCACGAAGA-AAGGAGCUCUu-5' miRNA<br>16 15 14 13 7 6 5 4 3 2<br>3'pairing Seed	CTCGAGA<br>7mer-m8		✗	M T

MiR-887-3p

CircRNA_102272

2D Structure	Local AU	Position	Conservation	Predicted By
34 7mer-m8 54<br>5'-caacacGA-GGCTGCCGTTCAg-3' UTR<br>3'-ggagccCUACCGCGGGCAAGUg-5' miRNA<br>16 15 14 13 7 6 5 4 3 2<br>3'pairing Seed	CCGTTCA<br>7mer-m8		✗	M T

Microarray analysis between Circ-RNA and Micro-RNA

(Based on Circ-RNA)

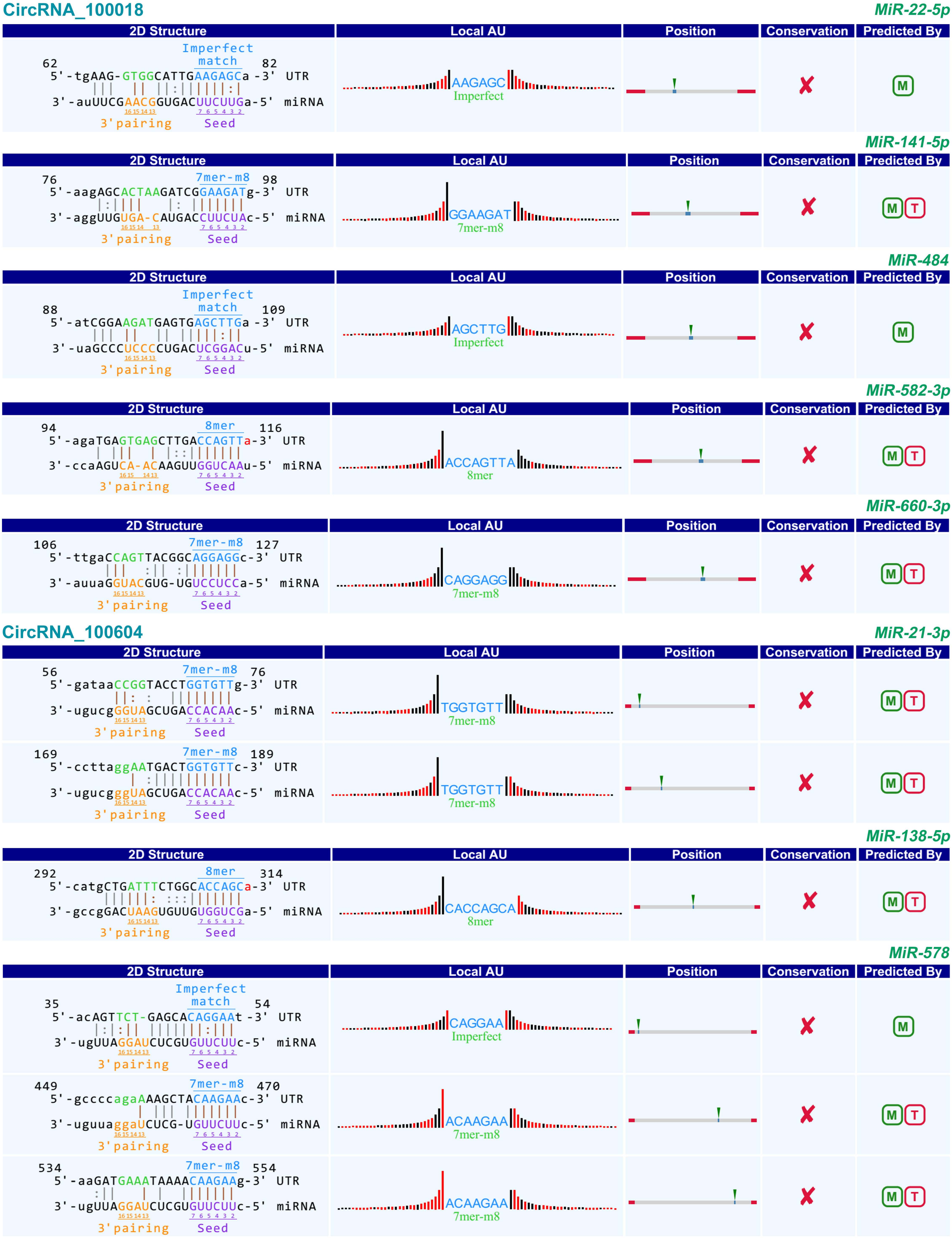

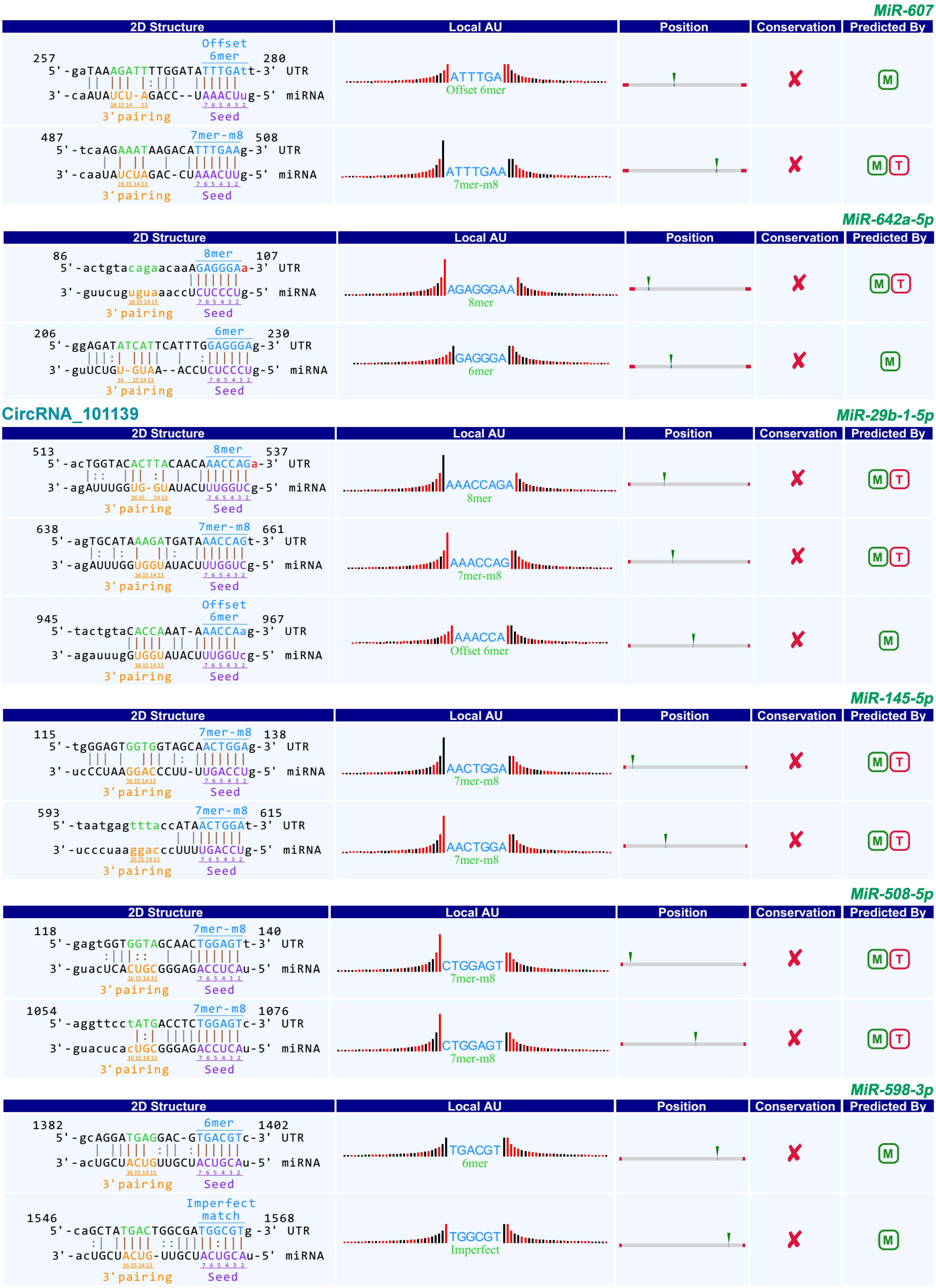
MiR-607
2D Structure
Local AU
Position
Conservation
Predicted By
257 Offset 6mer 280
5'-gaTAAAGATTTTGGATATTTGAtt-3' UTR
3'-caAUAUCU-AGACC--UAAACUug-5' miRNA
3'pairing Seed
ATTTGA
Offset 6mer
M
487 7mer-m8 508
5'-tcaAGAAATAAGACATTTGAAg-3' UTR
3'-caaUAUCUAGAC-CUAAACUUg-5' miRNA
3'pairing Seed
ATTTGAA
7mer-m8
M T
MiR-642a-5p
86 8mer 107
5'-actgtacagaacaaAGAGGGAa-3' UTR
3'-guucuguguaaaccUCUCCCUg-5' miRNA
3'pairing Seed
AGAGGGAA
8mer
M T
206 6mer 230
5'-ggAGATATCATTCATTTGGAGGGAg-3' UTR
3'-guUCUGU-GUAA--ACCUCUCCCUg-5' miRNA
3'pairing Seed
GAGGGA
6mer
M
CircRNA_101139
MiR-29b-1-5p
513 8mer 537
5'-acTGGTACACTTACAACAAACCAGa-3' UTR
3'-agAUUUGGUG-GUAUACUUUGGUCg-5' miRNA
3'pairing Seed
AAACCAGA
8mer
M T
638 7mer-m8 661
5'-agTGCATAAAGATGATAAACCAGt-3' UTR
3'-agAUUUGGUGGUAUACUUUGGUCg-5' miRNA
3'pairing Seed
AAACCAG
7mer-m8
M T
945 Offset 6mer 967
5'-tactgtaCACCAAAT-AAACCAag-3' UTR
3'-agauuugGUGGUAUACUUUGGUcg-5' miRNA
3'pairing Seed
AAACCA
Offset 6mer
M
MiR-145-5p
115 7mer-m8 138
5'-tgGGAGTGGTGGTAGCAACTGGAg-3' UTR
3'-ucCCUAAGGACCCUU-UGACCUg-5' miRNA
3'pairing Seed
AACTGGA
7mer-m8
M T
593 7mer-m8 615
5'-taatgagtttaccATAACTGGAt-3' UTR
3'-ucccuaaggaccCUUUGACCUg-5' miRNA
3'pairing Seed
AACTGGA
7mer-m8
M T
MiR-508-5p
118 7mer-m8 140
5'-gagtGGTGGTAGCAACTGGAGTt-3' UTR
3'-guacUCACUGCGGGAGACCUCAu-5' miRNA
3'pairing Seed
CTGGAGT
7mer-m8
M T
1054 7mer-m8 1076
5'-aggttcctATGACCTCTGGAGTc-3' UTR
3'-guacucacUGCGGGAGACCUCAu-5' miRNA
3'pairing Seed
CTGGAGT
7mer-m8
M T
MiR-598-3p
1382 6mer 1402
5'-gcAGGATGAGGAC-GTGACGTc-3' UTR
3'-acUGCUACUGUUGCUACUGCAu-5' miRNA
3'pairing Seed
TGACGT
6mer
M
1546 Imperfect match 1568
5'-caGCTATGACTGGCGATGGCGTg-3' UTR
3'-acUGCUACUG-UUGCUACUGCAu-5' miRNA
3'pairing Seed
TGGCGT
Imperfect
M

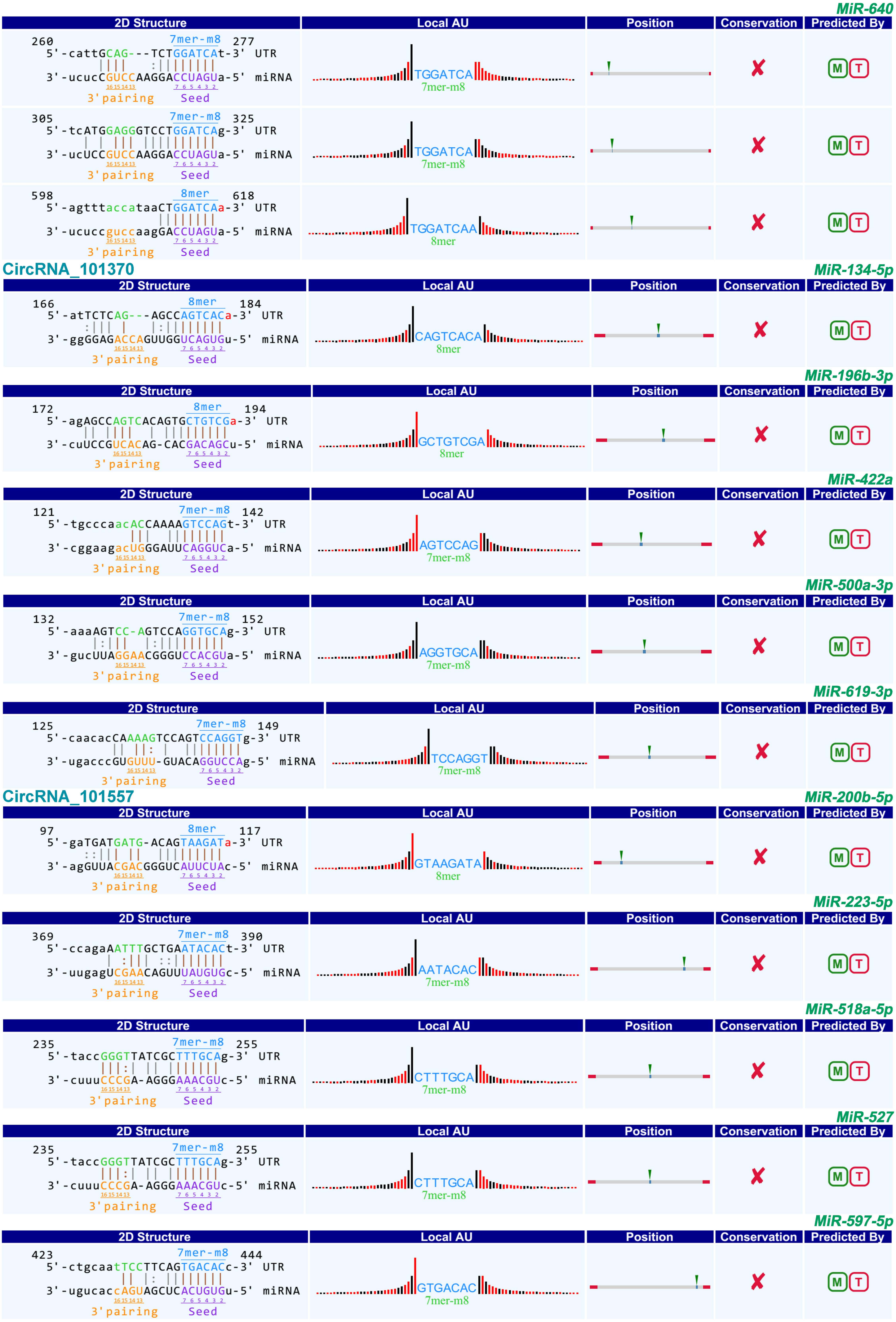
MiR-640
2D Structure
Local AU
Position
Conservation
Predicted By
260 7mer-m8 277
5'-cattGCAG---TCTGGATCAt-3' UTR
3'-ucucCGUCCAAGGACCUAGUa-5' miRNA
3'pairing Seed
TGGATCA
7mer-m8
305 7mer-m8 325
5'-tcATGGAGGGTCCTGGATCAg-3' UTR
3'-ucUCCGUCCAAGGACCUAGUa-5' miRNA
3'pairing Seed
TGGATCA
7mer-m8
598 8mer 618
5'-agtttaccataaCTGGATCAa-3' UTR
3'-ucuccguccaagGACCUAGUa-5' miRNA
3'pairing Seed
TGGATCAA
8mer
CircRNA_101370
MiR-134-5p
166 8mer 184
5'-atTCTCAG---AGCCAGTCACa-3' UTR
3'-ggGGAGACCAGUUGGUCAGUGu-5' miRNA
3'pairing Seed
CAGTCACA
8mer
MiR-196b-3p
172 8mer 194
5'-agAGCCAGTCACAGTGCTGTCGa-3' UTR
3'-cuUCCGUCACAG-CACGACAGCu-5' miRNA
3'pairing Seed
GCTGTCGA
8mer
MiR-422a
121 7mer-m8 142
5'-tgcccaacACCAAAAGTCCAGt-3' UTR
3'-cggaagacUGGGAUUCAGGUCa-5' miRNA
3'pairing Seed
AGTCCAG
7mer-m8
MiR-500a-3p
132 7mer-m8 152
5'-aaaAGTCC-AGTCCAGGTGCAg-3' UTR
3'-gucUUAGGAACGGGUCCACGUa-5' miRNA
3'pairing Seed
AGGTGCA
7mer-m8
MiR-619-3p
125 7mer-m8 149
5'-caacacCAAAGTCCAGTCCAGGTg-3' UTR
3'-ugacccGUGUUU-GUACAGGUCCAg-5' miRNA
3'pairing Seed
TCCAGGT
7mer-m8
CircRNA_101557
MiR-200b-5p
97 8mer 117
5'-gaTGATGATG-ACAGTAAGATa-3' UTR
3'-agGUUACGACGGGUCAUUCUAc-5' miRNA
3'pairing Seed
GTAAGATA
8mer
MiR-223-5p
369 7mer-m8 390
5'-ccagaAATTTGCTGAATACACt-3' UTR
3'-uugagUCGAACAGUUUAUGUGc-5' miRNA
3'pairing Seed
AATACAC
7mer-m8
MiR-518a-5p
235 7mer-m8 255
5'-taccGGGTTATCGCTTTGCAg-3' UTR
3'-cuuuCCCGA-AGGGAAACGUc-5' miRNA
3'pairing Seed
CTTTGCA
7mer-m8
MiR-527
235 7mer-m8 255
5'-taccGGGTTATCGCTTTGCAg-3' UTR
3'-cuuuCCCGA-AGGGAAACGUc-5' miRNA
3'pairing Seed
CTTTGCA
7mer-m8
MiR-597-5p
423 7mer-m8 444
5'-ctgcaatTCCTTCAGTGACACc-3' UTR
3'-ugucaccAGUAGCUCACUGUGu-5' miRNA
3'pairing Seed
GTGACAC
7mer-m8

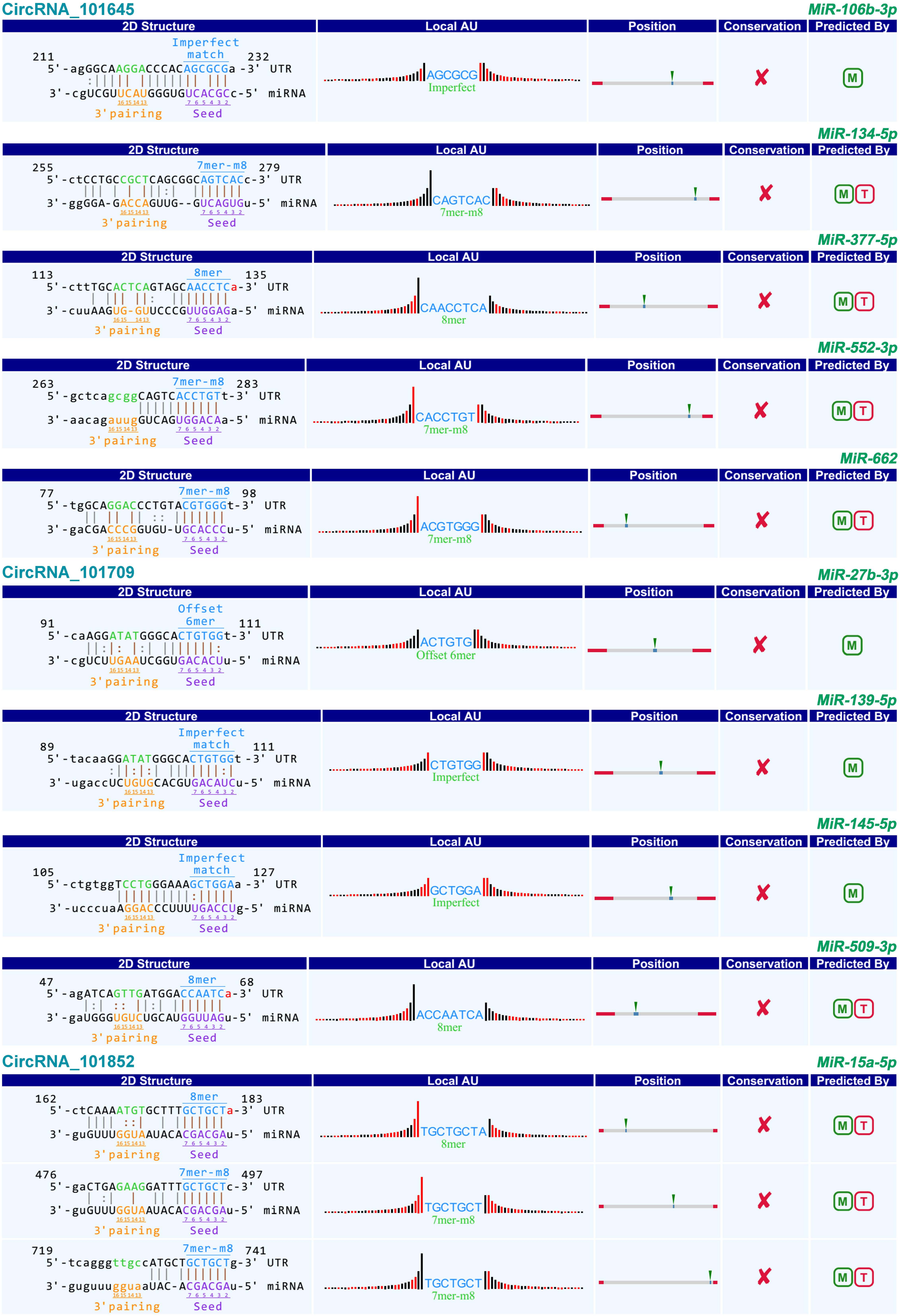

CircRNA_101645
MiR-106b-3p
2D Structure
Local AU
Position
Conservation
Predicted By
Imperfect match
211 232
5'-agGGCAAGGACCCACAGCGCGa-3' UTR
3'-cgUCGUUCAUGGGUGUCACGCc-5' miRNA
3'pairing Seed
AGCGCG
Imperfect
M
MiR-134-5p
7mer-m8
255 279
5'-ctCCTGCCGCTCAGCGGCAGTCACc-3' UTR
3'-ggGGA-GACCAGUUG--GUCAGUGu-5' miRNA
3'pairing Seed
CAGTCAC
7mer-m8
M T
MiR-377-5p
8mer
113 135
5'-cttTGCACTCAGTAGCAACCTCa-3' UTR
3'-cuuAAGUG-GUUCCCGUUGGAGa-5' miRNA
3'pairing Seed
CAACCTCA
8mer
M T
MiR-552-3p
7mer-m8
263 283
5'-gctcagcggCAGTCACCTGTt-3' UTR
3'-aacagauugGUCAGUGGACAa-5' miRNA
3'pairing Seed
CACCTGT
7mer-m8
M T
MiR-662
7mer-m8
77 98
5'-tgGCAGGACCCTGTACGTGGGt-3' UTR
3'-gaCGACCCGGUGU-UGCACCCu-5' miRNA
3'pairing Seed
ACGTGGG
7mer-m8
M T
CircRNA_101709
MiR-27b-3p
Offset 6mer
91 111
5'-caAGGATATGGGCACTGTGGt-3' UTR
3'-cgUCUUGAAUCGGUGACACUu-5' miRNA
3'pairing Seed
ACTGTG
Offset 6mer
M
MiR-139-5p
Imperfect match
89 111
5'-tacaaGGATATGGGCACTGTGGt-3' UTR
3'-ugaccUCUGUGCACGUGACAUCu-5' miRNA
3'pairing Seed
CTGTGG
Imperfect
M
MiR-145-5p
Imperfect match
105 127
5'-ctgtggTCCTGGGAAAGCTGGAa-3' UTR
3'-ucccuaAGGACCCUUUUGACCUg-5' miRNA
3'pairing Seed
GCTGGA
Imperfect
M
MiR-509-3p
8mer
47 68
5'-agATCAGTTGATGGACCAATCa-3' UTR
3'-gaUGGGUGUCUGCAUGGUUAGu-5' miRNA
3'pairing Seed
ACCAATCA
8mer
M T
CircRNA_101852
MiR-15a-5p
8mer
162 183
5'-ctCAAAATGTGCTTTGCTGCTa-3' UTR
3'-guGUUUGGUAAUACACGACGAu-5' miRNA
3'pairing Seed
TGCTGCTA
8mer
M T
7mer-m8
476 497
5'-gaCTGAGAAGGATTTGCTGCTc-3' UTR
3'-guGUUUGGUAAUACACGACGAu-5' miRNA
3'pairing Seed
TGCTGCT
7mer-m8
M T
7mer-m8
719 741
5'-tcagggttgccATGCTGCTGCTg-3' UTR
3'-guguuuggguaaUAC-ACGACGAu-5' miRNA
3'pairing Seed
TGCTGCT
7mer-m8
M T

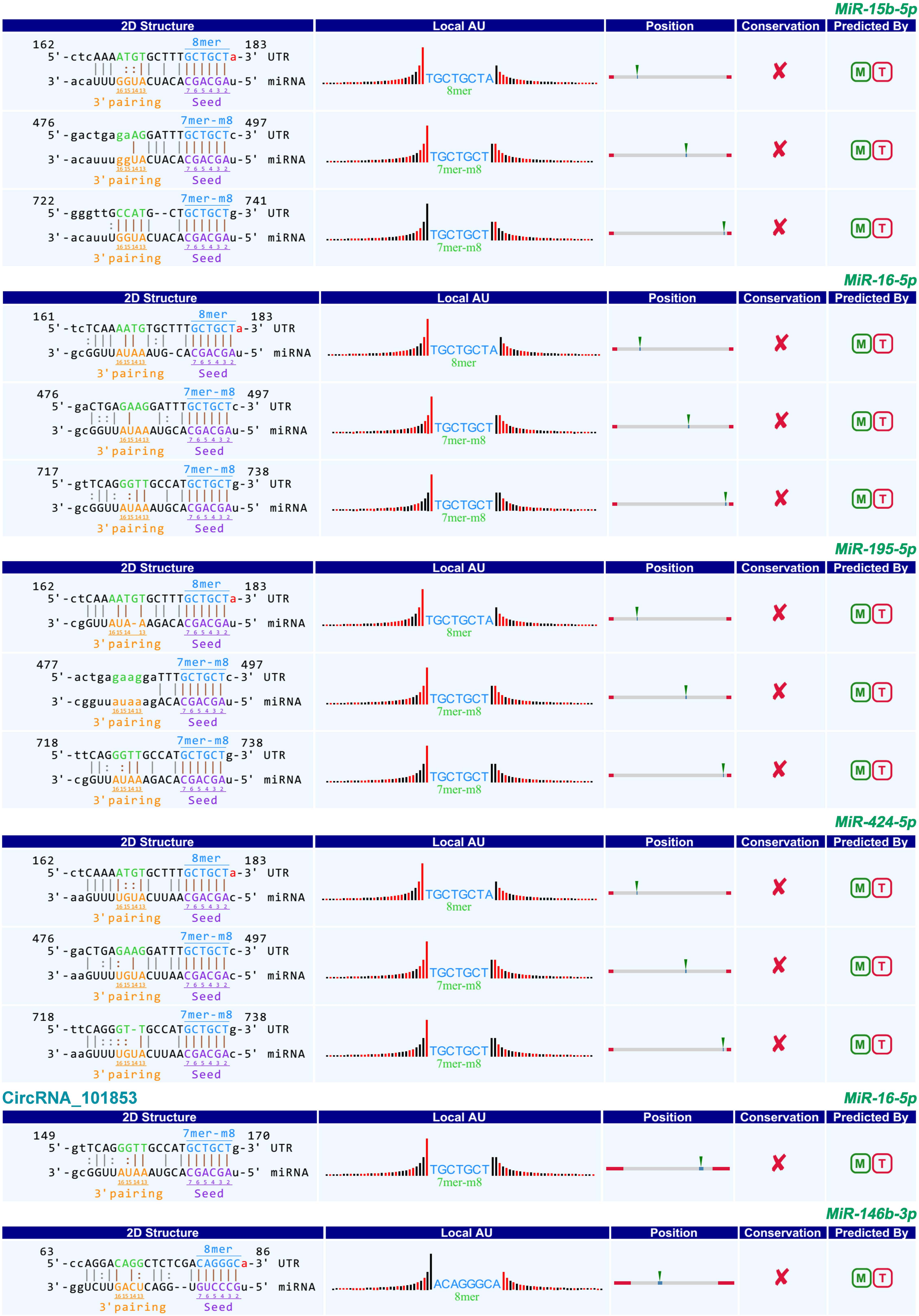
MiR-15b-5p
2D Structure
Local AU
Position
Conservation
Predicted By
162 8mer 183
5'-ctcAAAATGTGCTTTGCTGCTa-3' UTR
3'-acaUUUGGUACUACACGACGAu-5' miRNA
3'pairing Seed
TGCTGCTA
8mer
✗
M T
476 7mer-m8 497
5'-gactgagaAGGATTTGCTGCTc-3' UTR
3'-acauuuggUACUACACGACGAu-5' miRNA
3'pairing Seed
TGCTGCT
7mer-m8
✗
M T
722 7mer-m8 741
5'-gggttGCCATG--CTGCTGCTg-3' UTR
3'-acauuUGGUACUACACGACGAu-5' miRNA
3'pairing Seed
TGCTGCT
7mer-m8
✗
M T
MiR-16-5p
2D Structure
Local AU
Position
Conservation
Predicted By
161 8mer 183
5'-tcTCAAAATGTGCTTTGCTGCTa-3' UTR
3'-gcGGUUAUAAAUG-CACGACGAu-5' miRNA
3'pairing Seed
TGCTGCTA
8mer
✗
M T
476 7mer-m8 497
5'-gaCTGAGAAGGATTTGCTGCTc-3' UTR
3'-gcGGUUAUAAAUGCACGACGAu-5' miRNA
3'pairing Seed
TGCTGCT
7mer-m8
✗
M T
717 7mer-m8 738
5'-gtTCAGGGTTGCCATGCTGCTg-3' UTR
3'-gcGGUUAUAAAUGCACGACGAu-5' miRNA
3'pairing Seed
TGCTGCT
7mer-m8
✗
M T
MiR-195-5p
2D Structure
Local AU
Position
Conservation
Predicted By
162 8mer 183
5'-ctCAAAATGTGCTTTGCTGCTa-3' UTR
3'-cgGUUAUA-AAGACACGACGAu-5' miRNA
3'pairing Seed
TGCTGCTA
8mer
✗
M T
477 7mer-m8 497
5'-actgagaaggaTTTGCTGCTc-3' UTR
3'-cgguuauaaagACACGACGAu-5' miRNA
3'pairing Seed
TGCTGCT
7mer-m8
✗
M T
718 7mer-m8 738
5'-ttCAGGGTTGCCATGCTGCTg-3' UTR
3'-cgGUUAUAAGACACGACGAu-5' miRNA
3'pairing Seed
TGCTGCT
7mer-m8
✗
M T
MiR-424-5p
2D Structure
Local AU
Position
Conservation
Predicted By
162 8mer 183
5'-ctCAAAATGTGCTTTGCTGCTa-3' UTR
3'-aaGUUUUGUACUUAACGACGAc-5' miRNA
3'pairing Seed
TGCTGCTA
8mer
✗
M T
476 7mer-m8 497
5'-gaCTGAGAAGGATTTGCTGCTc-3' UTR
3'-aaGUUUUGUACUUAACGACGAc-5' miRNA
3'pairing Seed
TGCTGCT
7mer-m8
✗
M T
718 7mer-m8 738
5'-ttCAGGGT-TGCCATGCTGCTg-3' UTR
3'-aaGUUUUGUACUUAACGACGAc-5' miRNA
3'pairing Seed
TGCTGCT
7mer-m8
✗
M T
CircRNA_101853
MiR-16-5p
2D Structure
Local AU
Position
Conservation
Predicted By
149 7mer-m8 170
5'-gtTCAGGGTTGCCATGCTGCTg-3' UTR
3'-gcGGUUAUAAAUGCACGACGAu-5' miRNA
3'pairing Seed
TGCTGCT
7mer-m8
✗
M T
MiR-146b-3p
2D Structure
Local AU
Position
Conservation
Predicted By
63 8mer 86
5'-ccAGGACAGGCTCTCGACAGGGCa-3' UTR
3'-ggUCUUGACUCAGG--UGUCCCGu-5' miRNA
3'pairing Seed
ACAGGGCA
8mer
✗
M T

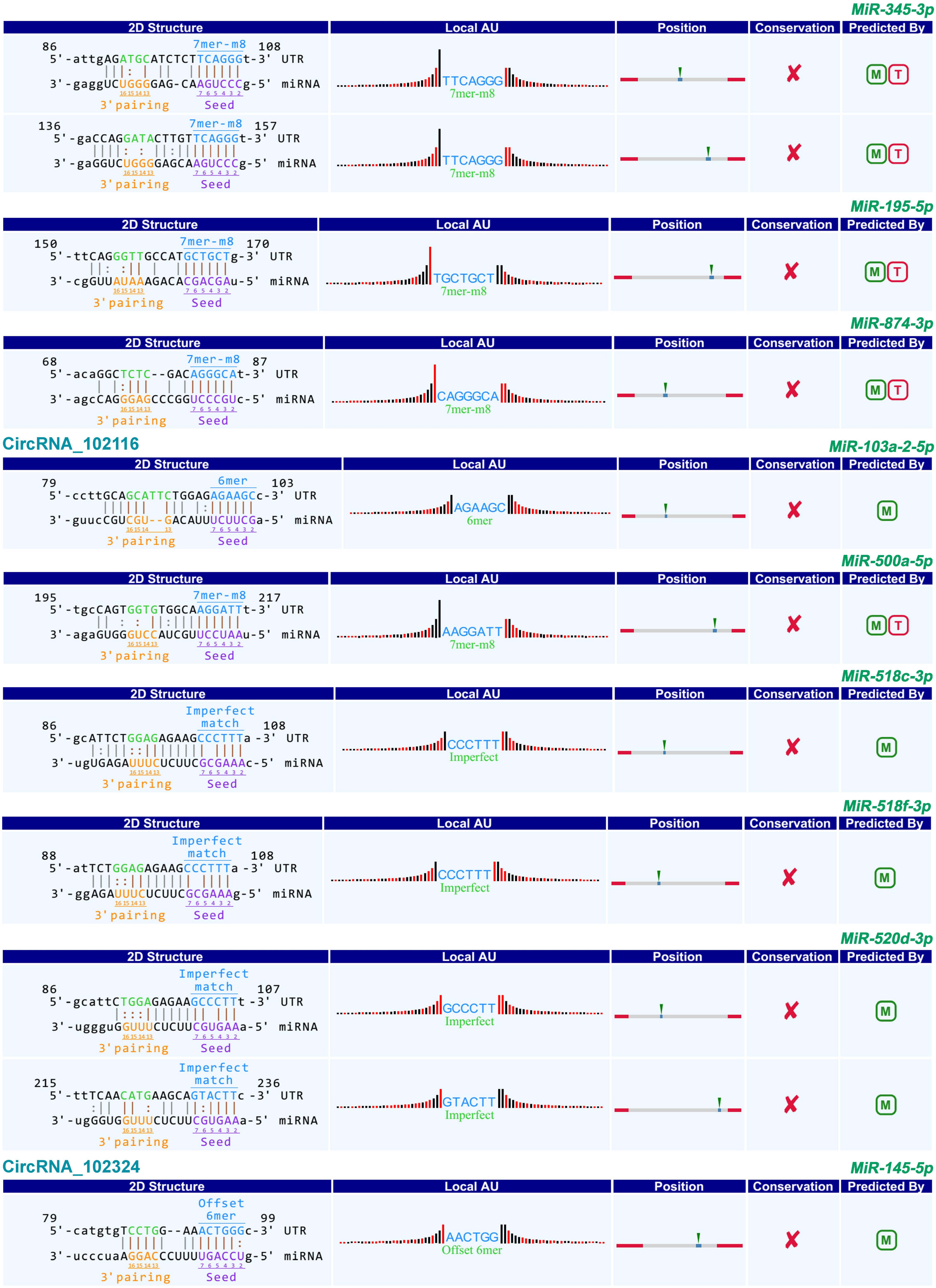

MiR-345-3p
2D Structure
Local AU
Position
Conservation
Predicted By
7mer-m8
86 108
5'-attgAGATGCATCTCTTCAGGGt-3' UTR
3'-gaggUCUGGGGAG-CAAGUCCCg-5' miRNA
3'pairing
Seed
TTCAGGG
7mer-m8
M T
7mer-m8
136 157
5'-gaCCAGGATACTTGTTCAGGGt-3' UTR
3'-gaGGUCUGGGGAGCAAGUCCCg-5' miRNA
3'pairing
Seed
TTCAGGG
7mer-m8
M T
MiR-195-5p
2D Structure
Local AU
Position
Conservation
Predicted By
7mer-m8
150 170
5'-ttCAGGGTTGCCATGCTGCTg-3' UTR
3'-cgGUUAUAAAGACACGACGAu-5' miRNA
3'pairing
Seed
TGCTGCT
7mer-m8
M T
MiR-874-3p
2D Structure
Local AU
Position
Conservation
Predicted By
7mer-m8
68 87
5'-acaGGCTCTC--GACAGGGCAt-3' UTR
3'-agcCAGGGAGCCCGGUCCCGUc-5' miRNA
3'pairing
Seed
CAGGGCA
7mer-m8
M T
CircRNA_102116
MiR-103a-2-5p
2D Structure
Local AU
Position
Conservation
Predicted By
6mer
79 103
5'-ccttGCAGCATTCTGGAGAGAAGCc-3' UTR
3'-guucCGUCGU--GACAUUUCUUCGa-5' miRNA
3'pairing
Seed
AGAAGC
6mer
M
MiR-500a-5p
2D Structure
Local AU
Position
Conservation
Predicted By
7mer-m8
195 217
5'-tgcCAGTGGTGTGGCAAGGATTt-3' UTR
3'-agaGUGGGUCCAUCGUUCCUAAu-5' miRNA
3'pairing
Seed
AAGGATT
7mer-m8
M T
MiR-518c-3p
2D Structure
Local AU
Position
Conservation
Predicted By
Imperfect match
86 108
5'-gcATTCTGGAGAGAAGCCCTTTa-3' UTR
3'-ugUGAGAUUUCUCUUCGCGAAAc-5' miRNA
3'pairing
Seed
CCCTTT
Imperfect
M
MiR-518f-3p
2D Structure
Local AU
Position
Conservation
Predicted By
Imperfect match
88 108
5'-atTCTGGAGAGAAGCCCTTTa-3' UTR
3'-ggAGAUUUCUCUUCGCGAAAg-5' miRNA
3'pairing
Seed
CCCTTT
Imperfect
M
MiR-520d-3p
2D Structure
Local AU
Position
Conservation
Predicted By
Imperfect match
86 107
5'-gcattCTGGAGAGAAGCCCTTt-3' UTR
3'-ugggguGGUUUCUCUUCGUGAAa-5' miRNA
3'pairing
Seed
GCCCTT
Imperfect
M
Imperfect match
215 236
5'-ttTCAACATGAAGCAGTACTTc-3' UTR
3'-ugGGUGGUUUCUCUUCGUGAAa-5' miRNA
3'pairing
Seed
GTACTT
Imperfect
M
CircRNA_102324
MiR-145-5p
2D Structure
Local AU
Position
Conservation
Predicted By
Offset 6mer
79 99
5'-catgtgTCCTGG--AAACTGGGc-3' UTR
3'-ucccuaAGGACCCUUUUGACCUg-5' miRNA
3'pairing
Seed
AACTGG
Offset 6mer
M

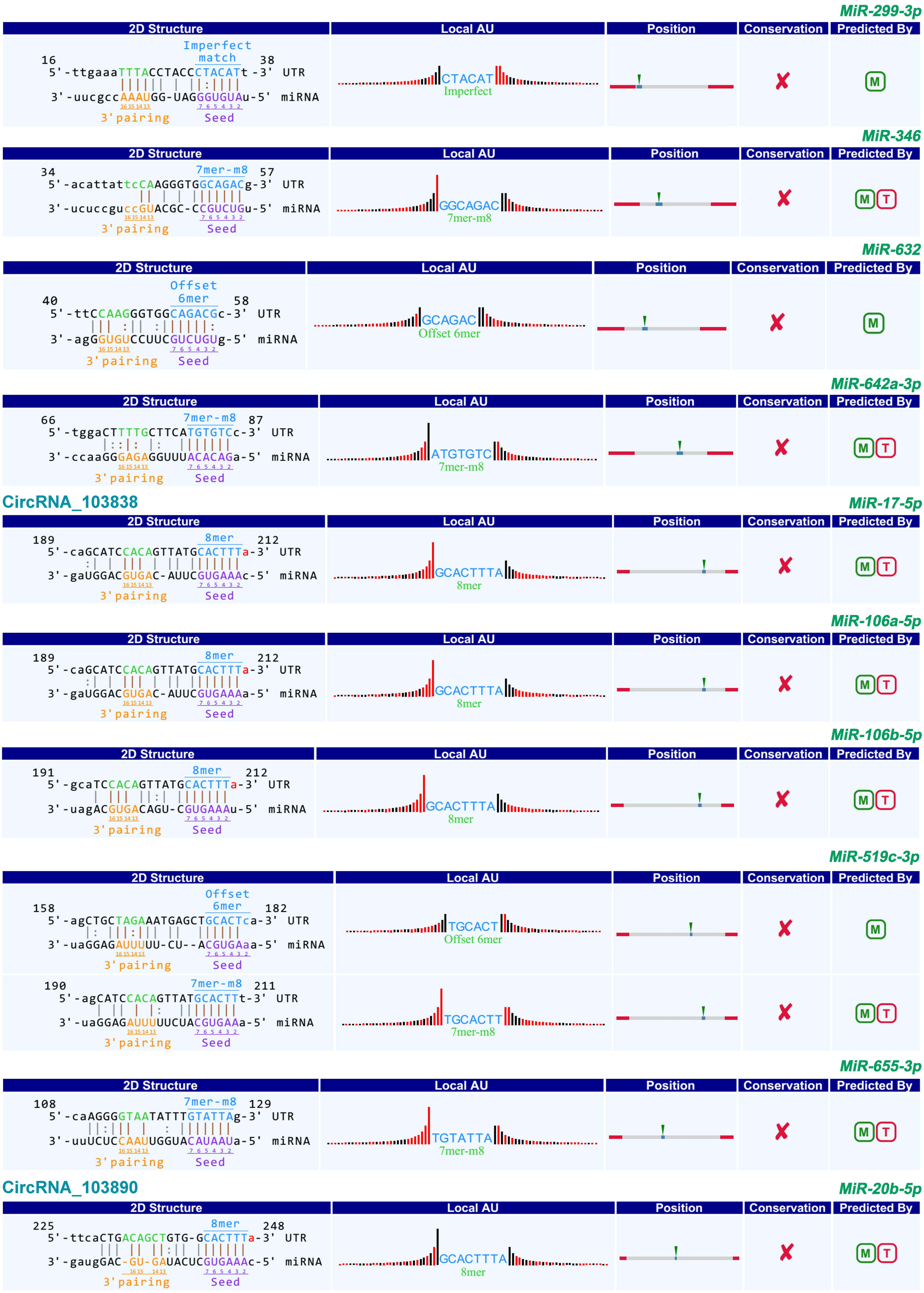
MiR-299-3p
2D Structure
Local AU
Position
Conservation
Predicted By
Imperfect match
16 38
5'-ttgaaaTTTACCTACCCTACATt-3' UTR
3'-uucgccAAAUGG-UAGGGUGUAu-5' miRNA
3'pairing
Seed
CTACAT
Imperfect
MiR-346
7mer-m8
34 57
5'-acattatctcCAAGGGTGGCAGACg-3' UTR
3'-ucuccguccGUACGC-CCGUCUGu-5' miRNA
GGCAGAC
7mer-m8
MiR-632
Offset 6mer
40 58
5'-ttCCAAGGGTGGCAGACGc-3' UTR
3'-agGGUGUCCUUCGUCUGUg-5' miRNA
GCAGAC
Offset 6mer
MiR-642a-3p
7mer-m8
66 87
5'-tggaCTTTTGCTTCATGTGTCc-3' UTR
3'-ccaaGGGAGAGGUUUACACAGa-5' miRNA
ATGTGTC
7mer-m8
CircRNA_103838
MiR-17-5p
8mer
189 212
5'-caGCATCCACAGTTATGCACTTTa-3' UTR
3'-gaUGGACGUGAC-AUUCGUGAAAc-5' miRNA
GCACTTTA
8mer
MiR-106a-5p
8mer
189 212
5'-caGCATCCACAGTTATGCACTTTa-3' UTR
3'-gaUGGACGUGAC-AUUCGUGAAAa-5' miRNA
GCACTTTA
8mer
MiR-106b-5p
8mer
191 212
5'-gcaTCCACAGTTATGCACTTTa-3' UTR
3'-uagACGUGACAGU-CGUGAAAu-5' miRNA
GCACTTTA
8mer
MiR-519c-3p
Offset 6mer
158 182
5'-agCTGCTAGAAATGAGCTGCACTca-3' UTR
3'-uaGGAGAUUUUU-CU--ACGUGAAa-5' miRNA
TGCACT
Offset 6mer
7mer-m8
190 211
5'-agCATCCACAGTTATGCACTTt-3' UTR
3'-uaGGAGAUUUUUCUACGUGAAa-5' miRNA
TGCACTT
7mer-m8
MiR-655-3p
7mer-m8
108 129
5'-caAGGGGTAATATTTGTATTAg-3' UTR
3'-uuUCUCCAAUUGGUACAUAAUa-5' miRNA
TGTATTA
7mer-m8
CircRNA_103890
MiR-20b-5p
8mer
225 248
5'-ttcaCTGACAGCTGTG-GCACTTTa-3' UTR
3'-gaugGAC-GU-GAUACUCGUGAAAc-5' miRNA
GCACTTTA
8mer

MiR-106b-5p

2D Structure	Local AU	Position	Conservation	Predicted By
227 8mer 248<br>5'-caCTGACAGCTGT-GGCACTTTa-3' UTR<br>3'-uaGAC-GU-GACAGUCGUGAAAu-5' miRNA<br>16 15 14 13 7 6 5 4 3 2<br>3'pairing Seed	GCACTTTA<br>8mer		✘	M T

MiR-185-5p

2D Structure	Local AU	Position	Conservation	Predicted By
Imperfect match<br>104 125<br>5'-atccaGAGTGTTTTTCTCTTCt-3' UTR<br>3'-aguccUUGACGGAAAGAGAGGu-5' miRNA<br>16 15 14 13 7 6 5 4 3 2<br>3'pairing Seed	CTCTTC<br>Imperfect		✘	M
Offset 6mer<br>473 493<br>5'-gtatccACTGTC-TTCTCTCat-3' UTR<br>3'-aguccuUGACGGAAAGAGAGgu-5' miRNA<br>16 15 14 13 7 6 5 4 3 2<br>3'pairing Seed	TCTCTC<br>Offset 6mer		✘	M

MiR-519c-3p

2D Structure	Local AU	Position	Conservation	Predicted By
Imperfect match<br>443 465<br>5'-ttCCTTTACAGAAGATGCGCTTg-3' UTR<br>3'-uaGGAGAU-UUUUCUACGUGAAa-5' miRNA<br>16 15 14 13 7 6 5 4 3 2<br>3'pairing Seed	GCGCTT<br>Imperfect		✘	M

MiR-583

2D Structure	Local AU	Position	Conservation	Predicted By
152 8mer 176<br>5'-caAATGTAGGCTTTGTACCTCTTTa-3' UTR<br>3'-caUUAC-CCUGGAA---GGAGAAAc-5' miRNA<br>16 15 14 13 7 6 5 4 3 2<br>3'pairing Seed	CCTCTTTA<br>8mer		✘	M T
321 7mer-m8 341<br>5'-ggaagcatATGTGCCTCTTTt-3' UTR<br>3'-cauuacccUGGAAGGAGAAAc-5' miRNA<br>16 15 14 13 7 6 5 4 3 2<br>3'pairing Seed	CCTCTTT<br>7mer-m8		✘	M T

CircRNA_104019

MiR-15b-5p

2D Structure	Local AU	Position	Conservation	Predicted By
Imperfect match<br>346 366<br>5'-acTACGCCG-GCTGTGCTGTTc-3' UTR<br>3'-acAUUUGGUACUACACGACGAu-5' miRNA<br>16 15 14 13 7 6 5 4 3 2<br>3'pairing Seed	GCTGTT<br>Imperfect		✘	M
554 8mer 574<br>5'-ttgctGCCGT-AGCTGCTGCTa-3' UTR<br>3'-acauuUGGUACUACACGACGAu-5' miRNA<br>16 15 14 13 7 6 5 4 3 2<br>3'pairing Seed	TGCTGCTA<br>8mer		✘	M T

MiR-361-5p

2D Structure	Local AU	Position	Conservation	Predicted By
Imperfect match<br>105 127<br>5'-tgACCCCAGGAACATTCTGTTAa-3' UTR<br>3'-caUGGGGACC-UCUAAGACUAUu-5' miRNA<br>16 15 14 13 7 6 5 4 3 2<br>3'pairing Seed	CTGTTA<br>Imperfect		✘	M
Imperfect match<br>258 280<br>5'-tggtgagaaGATGATTTTGATAg-3' UTR<br>3'-caugggggacCU-CUAAGACUAUu-5' miRNA<br>16 15 14 13 7 6 5 4 3 2<br>3'pairing Seed	TTGATA<br>Imperfect		✘	M

MiR-373-3p

2D Structure	Local AU	Position	Conservation	Predicted By
46 7mer-m8 71<br>5'-gaACCTCGATGGGATCAAAGCACTTt-3' UTR<br>3'-ugUGGGGUU---UUAGCUUCGUGAAg-5' miRNA<br>16 15 14 13 7 6 5 4 3 2<br>3'pairing Seed	AGCACTT<br>7mer-m8		✘	M T

MiR-424-3p

2D Structure	Local AU	Position	Conservation	Predicted By
321 8mer 341<br>5'-aggttgtatgaTGACGTTTTa-3' UTR<br>| |||||| 3'-uaucgucgcggAGUGCAAAAc-5' miRNA<br>16 15 14 13 7 6 5 4 3 2<br>3'pairing Seed	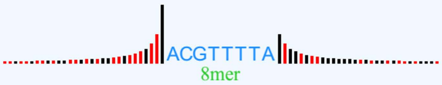		✗	M T
527 7mer-m8 547<br>5'-caccactgataggACGTTTTg-3' UTR<br>|||||| 3'-uaucgucgcggagUGCAAAAc-5' miRNA<br>16 15 14 13 7 6 5 4 3 2<br>3'pairing Seed	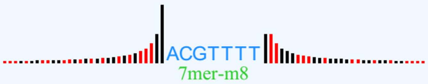		✗	M T

MiR-563

2D Structure	Local AU	Position	Conservation	Predicted By
Imperfect match<br>111 130<br>5'-caGGAACATTCTGTTAACCa -3' UTR<br>|:||| | |||:|||| 3'-ccCUUUG-CAUACAGUUGGa-5' miRNA<br>16 15 14 13 7 6 5 4 3 2<br>3'pairing Seed	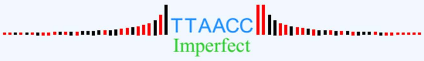		✗	M
Imperfect match<br>278 295<br>5'-taGGAA-GAATGTCAGCCc -3' UTR<br>|:|| | ||||||:|| 3'-ccCUUUGCAUACAGUUGGa-5' miRNA<br>16 15 14 13 7 6 5 4 3 2<br>3'pairing Seed	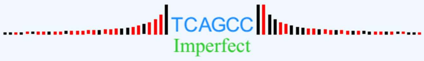		✗	M

CircRNA_104508

MiR-345-5p

2D Structure	Local AU	Position	Conservation	Predicted By
124 7mer-m8 145<br>5'-ggaTCCTGTGCTCTGAGTCAGc-3' UTR<br>:|||| :|| |||||| 3'-cucGGGACCUGAUCCUCAGUCg-5' miRNA<br>16 15 14 13 7 6 5 4 3 2<br>3'pairing Seed	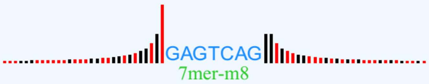		✗	M T

MiR-450b-5p

2D Structure	Local AU	Position	Conservation	Predicted By
Offset 6mer<br>89 111<br>5'-taTTGAAGAGGCATATTGCAATc-3' UTR<br>|| | || :|||||||||| 3'-auAAGUCCU-UGUAUAACGUUuu-5' miRNA<br>16 15 14 13 7 6 5 4 3 2<br>3'pairing Seed	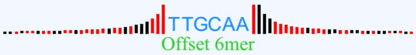		✗	M

MiR-488-3p

2D Structure	Local AU	Position	Conservation	Predicted By
139 7mer-m8 158<br>5'-agTCAGCCAGTA-CCTTTCAc-3' UTR<br>:||: |:|| |||||| 3'-cuGGUUCUUUAUCGGAAAGUu-5' miRNA<br>16 15 14 13 7 6 5 4 3 2<br>3'pairing Seed	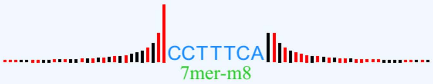		✗	M T

MiR-595

2D Structure	Local AU	Position	Conservation	Predicted By
233 7mer-m8 251<br>5'-cacCAAACCTC--CACACTTc-3' UTR<br>|| ||| | |||||| 3'-ucuGUGUGGUGCCGUGUGAAg-5' miRNA<br>16 15 14 13 7 6 5 4 3 2<br>3'pairing Seed	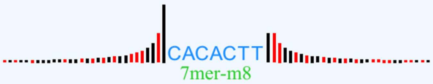		✗	M T

MiR-770-5p

2D Structure	Local AU	Position	Conservation	Predicted By
257 8mer 282<br>5'-cacCACTGACTGGATTTGGTACTGGa-3' UTR<br>| ||||| |: |||||||| 3'-accGGGACUG---UGCACCAUGACCu-5' miRNA<br>16 15 14 13 7 6 5 4 3 2<br>3'pairing Seed	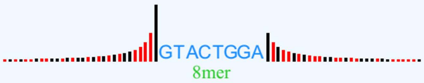		✗	M T

CircRNA_104600

MiR-452-3p

2D Structure	Local AU	Position	Conservation	Predicted By
123 7mer-m8 144<br>5'-ggCTTGCTGGCTAT-CAGATGAg-3' UTR<br>|||:|| || ||||||| 3'-guGAAUGA-AGAAACGUCUACUc-5' miRNA<br>16 15 14 13 7 6 5 4 3 2<br>3'pairing Seed	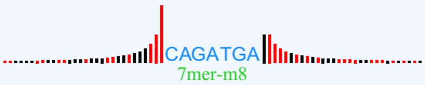		✗	M T

MiR-548c-3p

2D Structure	Local AU	Position	Conservation	Predicted By
57 7mer-m8 78<br>5'-gtAATGTTGATATAGATTTTTc-3' UTR<br>|| : |:|| ||||||| 3'-cgUUUUCAUUAACUCUAAAAAc-5' miRNA<br>16 15 14 13 7 6 5 4 3 2<br>3'pairing Seed	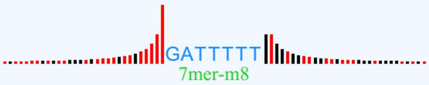		✗	M T

MiR-592

2D Structure	Local AU	Position	Conservation	Predicted By
133 7mer-m8 155<br>5'-ctATCAGATGAGTTTTGACACAg-3' UTR<br>3'-ugUAGU-AGCGUAUAACUGUGUu-5' miRNA<br>3'pairing Seed	TGACACA<br>7mer-m8		✘	M T

MiR-609

2D Structure	Local AU	Position	Conservation	Predicted By
Imperfect match<br>274 293<br>5'-tgAGAAGATTGAAACATCCa -3' UTR<br>3'-ucUCUACUCUCUUUGUGGGa-5' miRNA<br>3'pairing Seed	ACATCC<br>Imperfect		✘	M
308 7mer-m8 330<br>5'-acAGCTGGGAGTAACAACACCCg-3' UTR<br>3'-ucUCUACUCUC--U-UUGUGGGa-5' miRNA<br>3'pairing Seed	AACACCC<br>7mer-m8		✘	M T

MiR-758-5p

2D Structure	Local AU	Position	Conservation	Predicted By
69 7mer-m8 91<br>5'-tagatTTTCTGGACCAACCATc-3' UTR<br>3'-cacacGAGAGACC-AGUUGGUAg-5' miRNA<br>3'pairing Seed	CAACCAT<br>7mer-m8		✘	M T

CircRNA_104630

MiR-224-5p

2D Structure	Local AU	Position	Conservation	Predicted By
Imperfect match<br>163 183<br>5'-atTGGAAATAAAAGTGATTTa -3' UTR<br>3'-uuGCCUUGGUGAUCACUGAAc-5' miRNA<br>3'pairing Seed	TGATTT<br>Imperfect		✘	M

MiR-410-5p

2D Structure	Local AU	Position	Conservation	Predicted By
90 7mer-m8 112<br>5'-tacATTCAACCACTTGACAACCt-3' UTR<br>3'-gcuUGAG-UAGUG-UCUGUUGGa-5' miRNA<br>3'pairing Seed	GACAACC<br>7mer-m8		✘	M T

MiR-494-5p

2D Structure	Local AU	Position	Conservation	Predicted By
88 7mer-m8 112<br>5'-gatacattCAACCACTTGACAACCt-3' UTR<br>3'-ucucuucuGUU-GUG-CCUGUUGGa-5' miRNA<br>3'pairing Seed	GACAACC<br>7mer-m8		✘	M T

MiR-627-3p

2D Structure	Local AU	Position	Conservation	Predicted By
Imperfect match<br>181 200<br>5'-ttaGAATCTAGAAGAGAAGt -3' UTR<br>3'-ucaCUCAGAGUUUCUUUUCu-5' miRNA<br>3'pairing Seed	GAGAAG<br>Imperfect		✘	M

MiR-889-3p

2D Structure	Local AU	Position	Conservation	Predicted By
61 7mer-m8 80<br>5'-ttACTAGTT-TACGATATTAc-3' UTR<br>3'-ugUUACCAACAGGCUAUAAUu-5' miRNA<br>3'pairing Seed	GATATTA<br>7mer-m8		✘	M T

CircRNA_104703

MiR-147a

2D Structure	Local AU	Position	Conservation	Predicted By
264 7mer-m8 283<br>5'-agggctGCAATCCCACACAt-3' UTR<br>3'-cgucuuCGUAAAGGUGUGUg-5' miRNA<br>3'pairing Seed	CCACACA<br>7mer-m8		✘	M T

MiR-200b-3p

2D Structure	Local AU	Position	Conservation	Predicted By
300 7mer-m8 321<br>5'-aagtgcaaACCATTCAGTATTc-3' UTR<br>3'-aguaguaaUGGUCCGUCAUAAu-5' miRNA<br>3'pairing Seed	CAGTATT<br>7mer-m8		✘	M T

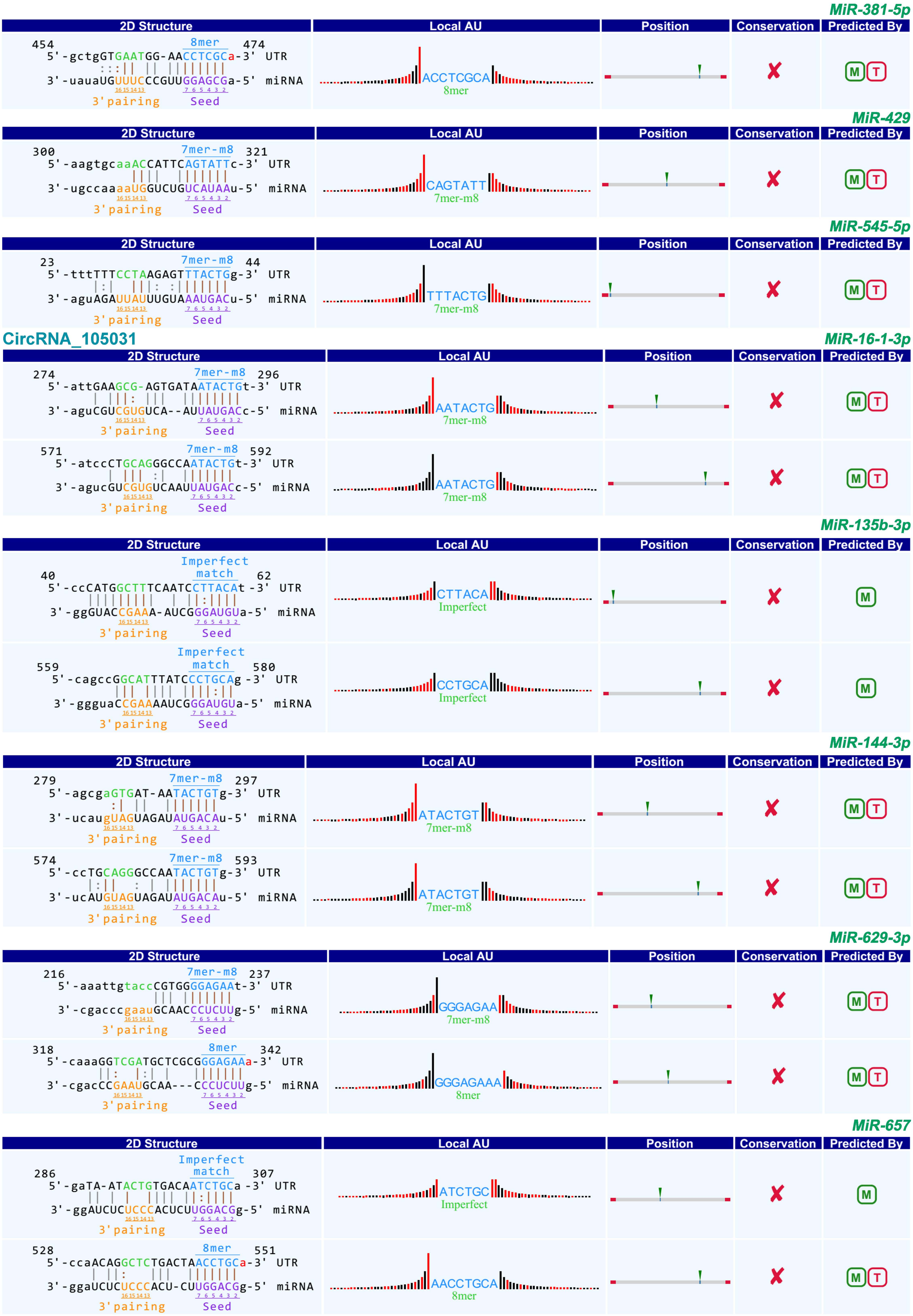

MiR-381-5p
2D Structure
Local AU
Position
Conservation
Predicted By
454 8mer 474
5'-gctgGTGAATGG-AACCTCGCa-3' UTR
3'-uauaUGUUUCCGUUGGAGCGa-5' miRNA
3'pairing Seed
ACCTCGCA
8mer
MiR-429
300 7mer-m8 321
5'-aagtgcaaACCATTCAGTATTc-3' UTR
3'-ugccaaaaUGGUCUGUCAUAAu-5' miRNA
3'pairing Seed
CAGTATT
7mer-m8
MiR-545-5p
23 7mer-m8 44
5'-tttTTTCCTAAGAGTTTACTGg-3' UTR
3'-aguAGAUUAUUUGUAAAUGACu-5' miRNA
3'pairing Seed
TTTACTG
7mer-m8
CircRNA_105031
MiR-16-1-3p
274 7mer-m8 296
5'-attGAAGCG-AGTGATAATACTGt-3' UTR
3'-aguCGUCGUGUCA--AUUAUGACc-5' miRNA
3'pairing Seed
AATACTG
7mer-m8
571 7mer-m8 592
5'-atccCTGCAGGGCCAATACTGt-3' UTR
3'-agucGUCGUGUCAAUUAUGACc-5' miRNA
3'pairing Seed
AATACTG
7mer-m8
MiR-135b-3p
40 Imperfect match 62
5'-ccCATGGCTTTCAATCCTTACAt-3' UTR
3'-ggGUACCGAAA-AUCGGGAUGUa-5' miRNA
3'pairing Seed
CTTACA
Imperfect
559 Imperfect match 580
5'-cagccGGCATTTATCCCTGCAg-3' UTR
3'-ggguaCCGAAAAUCGGGAUGUa-5' miRNA
3'pairing Seed
CCTGCA
Imperfect
MiR-144-3p
279 7mer-m8 297
5'-agcgaGTGAT-AATACTGTg-3' UTR
3'-ucaugUAGUAGAUAUGACAu-5' miRNA
3'pairing Seed
ATACTGT
7mer-m8
574 7mer-m8 593
5'-ccTGCAGGGCCAATACTGTg-3' UTR
3'-ucAUGUAGUAGAUAUGACAu-5' miRNA
3'pairing Seed
ATACTGT
7mer-m8
MiR-629-3p
216 7mer-m8 237
5'-aaattgtaccCGTGGGGAGAAt-3' UTR
3'-cgacccgaauGCAACCCUCUUg-5' miRNA
3'pairing Seed
GGGAGAA
7mer-m8
318 8mer 342
5'-caaaGGTCGATGCTCGCGGGAGAAa-3' UTR
3'-cgacCCGAAUGCAA---CCCUCUUg-5' miRNA
3'pairing Seed
GGGAGAAA
8mer
MiR-657
286 Imperfect match 307
5'-gaTA-ATACTGTGACAATCTGCa-3' UTR
3'-ggAUCUCUCCCACUCUUGGACGg-5' miRNA
3'pairing Seed
ATCTGC
Imperfect
528 8mer 551
5'-ccaACAGGCTCTGACTAACCTGCa-3' UTR
3'-ggaUCUCUCCCACU-CUUGGACGg-5' miRNA
3'pairing Seed
AACCTGCA
8mer
M T

CircRNA_400019

MiR-20b-3p

2D Structure	Local AU	Position	Conservation	Predicted By

```
              Imperfect
                match     216
195
 5'-catttcGTGCTTGAACTATAGc -3'  UTR
       ||||:::  ||||:||
 3'-gaccuuCACGGGUAUGAUGUCa-5'  miRNA
          16151413    765432
     3'pairing       Seed
```
Local AU: CTATAG Imperfect — Conservation: ✗ — Predicted By: M

```
731              7mer-m8   754
 5'-cacGCAGTTGGTACCTACTACAGt-3'  UTR
     | |||  |:  | |||||||
 3'-gacCUUCA--CGGGUAUGAUGUCa-5'  miRNA
         1615  1413    765432
     3'pairing         Seed
```
Local AU: ACTACAG 7mer-m8 — Conservation: ✗ — Predicted By: M T

MiR-135a-3p

2D Structure	Local AU	Position	Conservation	Predicted By

```
131              7mer-m8   151
 5'-taTCATAGGT-AGTTCCCTATc-3'  UTR
     :||: | |  : ||||||
 3'-gcGGUGCCGAGGUUAGGGAUAu-5'  miRNA
          16151413    765432
     3'pairing       Seed
```
Local AU: TCCCTAT 7mer-m8 — Conservation: ✗ — Predicted By: M T

```
              Imperfect
                match     372
351
 5'-tgcgggGGTTCTGGTCCCTGTg -3'  UTR
          ||:||:::|||||:|
 3'-gcggugCCGAGGUUAGGGAUAu-5'  miRNA
          16151413    765432
     3'pairing       Seed
```
Local AU: CCCTGT Imperfect — Conservation: ✗ — Predicted By: M

MiR-204-3p

2D Structure	Local AU	Position	Conservation	Predicted By

```
72               7mer-m8   93
 5'-tatataaTCTGTACTTCCCAGt-3'  UTR
          | ||:  |||||||
 3'-ugcagggAAACG-GAAGGGUCg-5'  miRNA
          16151413    765432
     3'pairing       Seed
```
Local AU: TTCCCAG 7mer-m8 — Conservation: ✗ — Predicted By: M T

```
430              7mer-m8   450
 5'-gctttaaaTCACTTCCCAGc-3'  UTR
          ||  |||||||
 3'-ugcagggaAACGGAAGGGUCg-5'  miRNA
          16151413    765432
     3'pairing       Seed
```
Local AU: TTCCCAG 7mer-m8 — Conservation: ✗ — Predicted By: M T

MiR-612

2D Structure	Local AU	Position	Conservation	Predicted By

```
156                   7mer-m8   180
 5'-gcaGTGCCTACTGTCACTGCCCAGg-3'  UTR
      | || :|  : | |||||||
 3'-uucCUCGAGUCUUCGGGACGGGUCg-5'  miRNA
             16151413    765432
        3'pairing       Seed
```
Local AU: TGCCCAG 7mer-m8 — Conservation: ✗ — Predicted By: M T

```
633                                8mer      665
 5'-tgGGGGCAGTCGGTGCCCCCGTTAGTGCCCAGa-3'  UTR
      ||:||  ||:| : |||     ||||||
 3'-uuCCUCG--AGUCUUCGGG------ACGGGUCg-5'  miRNA
              16151413         765432
        3'pairing              Seed
```
Local AU: TGCCCAGA 8mer — Conservation: ✗ — Predicted By: M T

```
                   Imperfect
                     match     691
667
 5'-caGAAACATACATACCCTGCCTAGg -3'  UTR
      | | | :| |  |||||||:||
 3'-uuCCUCGAGUCUUCGGGACGGGUCg-5'  miRNA
             16151413    765432
        3'pairing       Seed
```
Local AU: GCCTAG Imperfect — Conservation: ✗ — Predicted By: M

MiR-1298-3p

2D Structure	Local AU	Position	Conservation	Predicted By

```
              Imperfect
                match     182
163
 5'-ctaCTGTCA-CTGCCCAGGTt -3'  UTR
      | ||||  ||||||:|
 3'-caaGUCAGUCAACGGGUCUAc-5'  miRNA
         16151413   765432
     3'pairing      Seed
```
Local AU: CCAGGT Imperfect — Conservation: ✗ — Predicted By: M

```
241             7mer-A1   262
 5'-ggccctTCATGTTCTCCAGATa-3'  UTR
          ||| ||| :|||||
 3'-caagucAGU-CAACGGGUCUAc-5'  miRNA
          16151413    765432
     3'pairing       Seed
```
Local AU: TCCAGATA 7mer-A1 — Conservation: ✗ — Predicted By: M T

```
648             7mer-m8   667
 5'-cccCCGTTAG-TGCCCAGATc-3'  UTR
      | ||:|| ||||||||
 3'-caaGUCAGUCAACGGGUCUAc-5'  miRNA
         16151413   765432
     3'pairing      Seed
```
Local AU: CCCAGAT 7mer-m8 — Conservation: ✗ — Predicted By: M T
